# Orchard management alters citrus root and rhizosphere microbiomes with functional consequences for plant performance

**DOI:** 10.1093/ismeco/ycag248

**Published:** 2026-09-02

**Authors:** Nichole Ginnan, Riley Jones, Jessica Wu-Woods, Tariq Pervaiz, Ashraf El-kereamy, Vanessa E T M Ashworth, Muhammad Imran Hamid, Emma K Dawson, Sarah L Strauss, Jason Stajich, Philippe Rolshausen, Caroline Roper

**Affiliations:** Department of Microbiology and Plant Pathology, University of California, Riverside, CA 92521, United States; Department of Botany and Plant Sciences, University of California, Riverside, CA 92521, United States; Department of Microbiology and Plant Pathology, University of California, Riverside, CA 92521, United States; Department of Botany and Plant Sciences, University of California, Riverside, CA 92521, United States; Department of Botany and Plant Sciences, University of California, Riverside, CA 92521, United States; Department of Botany and Plant Sciences, University of California, Riverside, CA 92521, United States; Department of Botany and Plant Sciences, University of California, Riverside, CA 92521, United States; Department of Soil, Water, and Ecosystems Sciences, Southwest Research and Education Center, University of Florida, Immokalee, FL 34142, United States; Department of Soil, Water, and Ecosystems Sciences, Southwest Research and Education Center, University of Florida, Immokalee, FL 34142, United States; Department of Microbiology and Plant Pathology, University of California, Riverside, CA 92521, United States; Department of Botany and Plant Sciences, University of California, Riverside, CA 92521, United States; Department of Microbiology and Plant Pathology, University of California, Riverside, CA 92521, United States

**Keywords:** plant microbiome, rhizosphere microbiome, agricultural management, microbiome function, plant–microbe interactions, citrus microbiome, soil microbiology, legacy effects, disturbance ecology

## Abstract

Agricultural management practices act as ecological disturbances that can restructure soil and plant-associated microbial communities, but the functional consequences of these microbial shifts on crop performance remain poorly understood. Here, we examined how common orchard inputs, including wood mulch, glyphosate, and humic acid, affect citrus root and rhizosphere microbiomes, leaf nutrient trajectories, and tree performance over a 3-year field experiment. Mulch emerged as the dominant driver of fungal community composition and taxonomic turnover, enriching for saprophytic fungi. Conversely, bacterial communities responded primarily to interactions among applications, particularly mulch × glyphosate, which was associated with significant depletions of bacterial genera in roots and rhizospheres. These microbiome changes corresponded with reduced tree carbon assimilation, transpiration, and yield, and altered leaf nutrients dynamics. To verify whether the microbial shifts were contributing to these plant phenotypic changes, we conducted a greenhouse experiment using field-derived soil microbiota. Active microbiota from mulch-treated soils reduced citrus seedling establishment and root growth relative to microbiota from nonmulched soils, whereas heat-killed controls eliminated these negative effects, demonstrating a causal relationship between management-induced microbiota changes and decreases in plant performance. The effect of mulch-associated microbiota on root growth further depended on glyphosate history, paralleling field observations. Humic acid increased root growth regardless of microbiota activity and moderated decreases in shoot growth by mulch-associated microbiota. Together, these results show that management practices can restructure citrus microbiomes and generate community-level traits that influence plant performance, highlighting the importance of incorporating microbial ecology and microbiome information when devising crop management strategies.

## Introduction

Growers are increasingly challenged to balance high productivity, rising input costs, and environmental impacts to maintain profitable and sustainable cropping systems [1]. Achieving this balance requires agricultural practices that work with, rather than against, the ecological processes that support plant growth. Historically, most management strategies were developed with limited to no consideration of soil microbial communities. However, it is now clear that soil- and plant-associated microbiomes play vital roles in regulating plant nutrition, mediating stress tolerance, and suppressing pathogens [2–7]. Agricultural inputs such as herbicides, organic amendments, and ground cover management alter plant and soil microbial communities [8]. Understanding how management practices influence microbiome composition and function is a critical step towards developing approaches to steer microbial communities to sustainably enhance plant performance.

Management practices can influence plant performance directly and indirectly by modifying microbiome functions and plant–microbe interactions. For example, field amendments and cover crops can alter plant root exudates, which impacts plant microbiome assembly [9–12]. From an ecological perspective, many agricultural inputs function as disturbances that alter microbial community composition, diversity, and functional potential [13, 14]. While the negative impacts of mechanical disturbances, like tillage, have been well characterized [15–17], the effects of commonly used herbicides and organic inputs on microbial community form and function are less well understood, particularly for perennial tree crops.

Glyphosate-based herbicides are widely used to manage weeds in agricultural systems. Glyphosate blocks the activity of 5-enolpyruvylskhikimate-3-phosphate synthase, an enzyme in the shikimate pathway, which is essential for the synthesis of important aromatic amino acids [18, 19]. In addition to its herbicidal activity, glyphosate can alter soil, plant, and animal gut microbial communities, as many bacteria and fungi also possess the targeted pathway [18, 20–22]. Previous studies have reported glyphosate-induced reductions in microbial diversity and plant growth–promoting taxa, as well as increases in glyphosate-degrading microorganisms [20]. For example, glyphosate can decrease arbuscular mycorrhizal fungal colonization of roots [23] and increase abundance of *Fusarium* spp. [24, 25]. Because glyphosate is commonly applied alongside fertilizers and other amendments, interactions among these inputs may further complicate their effects on soil microbial communities and plant performance.

Organic amendments also influence soil properties and microbial communities [26, 27]. Wood mulch is often applied in perennial cropping systems to suppress weeds and retain soil moisture [28, 29]. Mulch can also alter soil nutrient availability and has been associated with increased microbial diversity and shifts in rhizosphere community composition [30]. Similarly, humic acid (HA), an organic component of soil organic matter, can stimulate plant growth and root development, through direct modulation of plant physiology [31, 32] and indirect microbial community alterations, such as enrichment of pathogen-suppressive bacteria [33]. Furthermore, organic amendments, such as earthworm cast, can decrease glyphosate phytotoxicity when applied in combination [34], again highlighting the need to study the combined effects of herbicide and organic amendments on plant-associated microbiota.

Citrus is an important global tree crop and has emerged as a model system for studying plant–microbiota interactions in perennial agriculture [35]. Citrus trees harbor diverse microbial communities across root, rhizosphere, and aboveground tissues [36]. These microbiomes vary across plant compartments, host genotypes, and geographic locations, while still maintaining a core set of microbial taxa [36–40]. Shifts in citrus-associated microbiomes have been linked to plant developmental stage, disease progression, and seasonality [41–43]. Moreover, the citrus microbiome is a reservoir of microbial taxa with potential to help control citrus pathogens, understand disease holistically, and promote growth and fruit quality [44–49].

Despite the growing recognition that management practices influence crop microbiomes, it remains difficult to determine whether these changes translate into meaningful functional effects on plant performance. Microbial kingdoms (bacteria, fungi, archaea) often have different responses, and while studies have revealed associations between management practices, microbiome composition, and plant phenotypes, experimental validation is needed to establish causal relationships [50].

In this study, we investigated how common orchard management practices influenced citrus microbiomes and tree performance over three years. Field plots received combinations of wood mulch, glyphosate, and HA applications in a factorial design. We first evaluated the effects of these treatments on tree nutrient status and performance, weed management, and root and rhizosphere bacterial and fungal communities in the field. Then, to test whether management-driven microbiome shifts persist and translate into functional consequences for plant growth, we conducted a greenhouse experiment where soil microbiota from the field plots were inoculated into sterile substrate and used to propagate citrus rootstock seedlings. By comparing active microbial inocula with heat-killed controls, we distinguished microbial effects from abiotic soil effects. Together, these experiments determined that common orchard management practices do indeed alter microbiome community-level traits in ways that influence citrus phenotypes.

## Materials and methods

### Field experiment site and design

**Figure 1:**
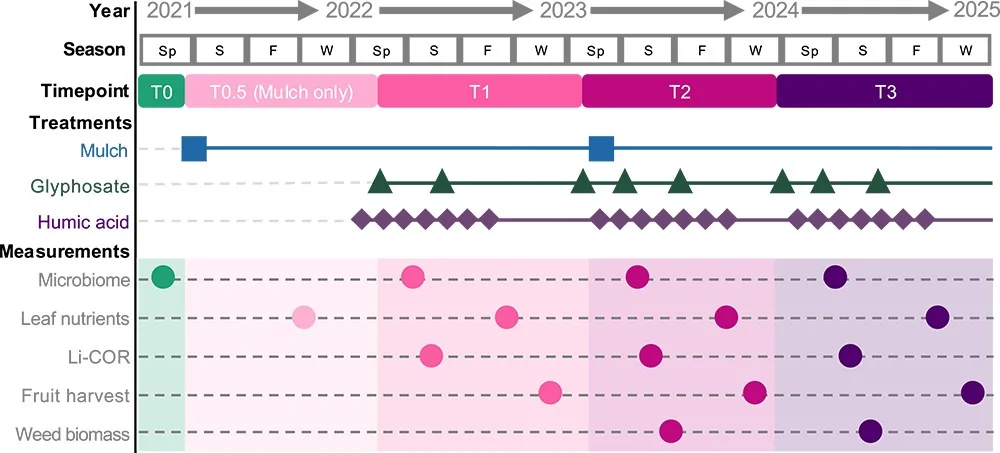
Experimental and sampling timeline. The timeline indicates the year (2021–5), season (Sp = Spring, S = Summer, F = Fall, W = Winter), and the experimental timepoint (T0–T3) of specific treatments and measurements. Application events are represented by blue squares (wood mulch), green triangles (glyphosate), and purple diamonds (humic acid, HA). Circles represent sampling or data collection events, including root and rhizosphere microbiome samples (microbiome), leaves for nutrients analysis (leaf nutrients), leaf carbon assimilation and transpiration rates measurements using the Li-COR LI-6800 portable photosynthesis system (Li-COR), fruit collection for counts and weighing (fruit harvest), and aboveground weed tissue collection (weed biomass). The circle colors correspond to the experimental timepoint they represent.

The field experiment was conducted from April 2021 to March 2025 (Fig. 1) at the Lindcove Research and Extension Center (LREC) in Exeter, CA, USA. Eight treatment plots (16 trees/plot; 4 × 4 format) containing “Tango” mandarin orange (*Citrus reticulata* Blanco) trees grafted on Carrizo rootstock [*C. sinensis* (L.) Osbeck × *Poncirus trifoliata* (L.) Rafinesque] and planted in 2011 were established in a full factorial design crossing three management factors: wood mulch, glyphosate (herbicide), and HA (Table 1). Tree rows were spaced 5.5 m apart, with trees planted 3 m apart within rows. For sample collection, the border trees on each plot were not sampled to avoid edge effects. Because each treatment was applied at the plot level, a single mature orchard plot was used for each treatment combination. To account for pre-existing microbial differences among plots, all study trees were sampled before treatment application (pretreatment; timepoint zero, T0), and the same individual trees were subsequently sampled annually for 3 years, following the start of treatment (T1–T3; Fig. 1). This repeated-measures design enables treatment effects to be evaluated relative to each tree’s pretreatment microbiome.

**Table 1:** Field application treatment groups.

Treatment group ID	Mulch	Glyphosate	Humic acid
CCC	–	–	–
CCH	–	–	Yes
CGC	–	Yes	–
CGH	–	Yes	Yes
MCC	Yes	–	–
MCH	Yes	–	Yes
MGC	Yes	Yes	–
MGH	Yes	Yes	Yes

Initially, wood mulch consisted of chipped almond tree material sourced from a local nursery (Visalia, CA, USA). Mulch application was completed in May 2021 at 10 tons/acre (Fig. 1). Mulch was applied across the whole plot, including between rows and beneath canopies to a 7–8 cm depth (Supplementary Fig. S1). Mulch from chipped citrus trees originating from the LREC was reapplied in April 2023 at ~8 tons/acre.

Glyphosate (Roundup original, Bayer Crop Sciences, NC, USA) application started in 2022 and was applied in May and August, at 16 oz per 50 gallons of water (Fig. 1). Glyphosate was applied again in March, May, and August of 2023 and 2024. HA (Growth Boost, SoilBiotics, Kankakee, IL, USA) was applied under the tree canopy around the trunk at 5 gallons/acre monthly from April to October in 2022, 2023, and 2024. Afterward, application sprinklers were run for 30 min to facilitate product absorption into soil and roots.

All treatment plots received identical standard management practices both before and after the start of the applications. Irrigation was applied at 2-week intervals for 48 hours, totaling ~3.7 × 10^3^ m^3^ of water per year. Twenty units of calcium ammonium nitrate (17-0-0; CAN 17) were applied monthly from April to August.

### Data collection and field sampling

Samples were collected for leaf nutrient measurements in November each year (2021–4; T0.5, T1, T2, T3; Fig. 1). Fifteen leaves were collected from each quadrant of four replicate trees per plot. For each tree, the 60 leaves were pooled into a single representative sample in a plastic bag. Fresh leaf samples were shipped to the Fruit Growers Laboratory, Inc. (2021–2; Santa Paula, CA, USA) or Valley Tech Agricultural Labs (2023–4; Tulare, CA, USA) for a leaf comprehensive group nutrient analysis. Because companies were switched for T2–T3, we only make comparisons within years, not across years.

Photosynthetic measurements were collected each year in June (2022, 2023, 2024) on fully expanded sun-exposed leaves between 11:00 a.m. and 12:30 p.m., using a LI-6800 portable photosynthesis system (Li-Cor, Inc., Lincoln, NE, USA) (Fig. 1). Measurements were repeated on four west facing leaves (technical replicates) on each tree. Six replicate trees were sampled per treatment plot each year (*n* = 192).

Fruit was harvested between 28 February and 5 March of 2023, 2024, and 2025 (Fig. 1). The fruits from each tree were transported in bins to an on-site pack line where total fruit weight and fruit counts were collected. Average fruit weight was calculated by dividing the total weight by the number of fruits.

Weed aboveground biomass was collected in August 2023 and 2024 by dividing each plot by tree rows and collecting weeds from three replicate rows per treatment plot using a sickle (Fig. 1). Weed fresh weight was measured. Then, samples were dried for 2 months under ambient conditions and dry weight was measured.

### Microbiome sample collection and processing

Each year, including April 2021 (T0) and late May to early June 2022–4 (T1–T3), root and rhizosphere samples were collected (*n* = 128 tree samples; 4 biological replicates × 8 treatment groups × 4 time points) for microbiome analyses, again avoiding border trees (Fig. 1). Root samples were collected near the irrigation line of each tree by removing the top 5 cm layer and digging into the root zone 15–20 cm and collecting ~10 g of roots in 50 ml falcon tubes. Roots were washed twice with 25 ml of sterile diH_2_O by vigorously shaking the falcon tubes. Roots and liquids (rhizosphere samples) were then separated, flash-frozen in liquid nitrogen, and stored at −80°C. Because roots were only rinsed with sterile water, rather than surface-sterilized, our root microbial communities include microorganisms colonizing internal root tissues (endophytes) and the root surface (epiphytes).

Root tissue was lyophilized in a FreeZone 2.5 L benchtop freeze dryer (Labconco, Kansas City, USA) for 72 h. Lyophilized tissue was ground into a powder on a MM 400 mill (Retsch, Haan, Germany) in 35 ml stainless-steel grinding jars, each containing a 20 mm stainless-steel ball, at 25 oscillations/s delivered in 30-s increments.

### Microbial DNA extractions, library preparations, and amplicon sequencing

Rhizosphere samples (root wash liquid) were thawed and centrifuged at 4000 *g* for 8 min [51]. The supernatant was removed, and DNA was extracted from the pellet. Rhizosphere pellet and dried root powder DNA was extracted and purified using a high-throughput workflow implemented on a QIAcube HT robotic instrument (QIAGEN, Hilden, Germany) (Supplementary Methods S1). Amplicon sequencing libraries were prepared, following Earth Microbiome Project protocols [52, 53] (Supplementary Methods S2).

Library sequencing was completed by the UC Riverside Genomics Core Facility on an Illumina NextSeq (2000 P1 600 cycle kit). NextSeq 2000 runs provide up to 100 million reads per run, and ~450 samples were multiplexed per run for an average of 222 222 reads per sample.

### Amplicon sequence processing

Raw Illumina sequences were demultiplexed, with zero index barcode mismatches allowed, and converted to FASTQ format using BCL Convert v3.8.4. Adaptors and primer sequences were removed using trimmomatic v0.39 [54] with standard settings and cutadapt v5.0 [55] with a minimum 5-base overlap, maximum 15% error rate when matching primers, and used –nextseq-trim = 20 to trim polyX tails and low-quality ends. FastQC v0.12.1 [56] was used to verify read quality and check for adapter sequences. Additional quality control and reads processing were completed using the DADA2 v1.38.0 pipeline, and positive and negative control samples were assessed [57] (Supplementary Methods S3).

### Microbial community analyses

All analyses were completed in R (v4.5.0) [58]. Alpha diversity analysis methods are available in Supplementary Methods S4 and models are in Supplementary Table S1. Community beta diversity across timepoints (T0, T1, T2, and T3) and across treatment plots prior to treatment (T0) was assessed within each of the four communities examined with a constrained analysis of principal coordinates (CAP) of centered-log-ratio-transformed counts, using phyloseq::ordinate, method = “CAP” and distance = “Euclidean” with the following formulas, respectively:


\begin{align*} &\sim \mathrm{Timepoint} + \mathrm{Condition}(\mathrm{scale}(\mathrm{logObs})).\\&\sim \mathrm{Mulch}* \mathrm{Glyphosate} * \mathrm{Humic} + \mathrm{Condition}(\mathrm{scale}(\mathrm{logObs})). \end{align*}

An ANOVA-like permutation test (PERMANOVA) was used to test for model significance using the vegan::anova.cca() (v2.7-1) function (Supplementary Tables S2 and S3) [59]. CAP1 and CAP2 axes were plotted using ggplot2 v3.5.1, including 95% confidence interval ellipse (stat_ellipse()) [60, 61].

Additionally, a CAP was performed to test for differences in community composition among treatment plots (T1–T3) while accounting for baseline community variation (T0) across plots. To characterize baseline community variation, a partial ordination was first performed on the T0 samples while conditioning on sequencing depth. The first three ordination axes from this analysis were then included as conditioning variables in the CAP analysis of the T1–T3 samples, together with sequencing depth and sampling timepoint, using the following model:


\begin{align*} & \sim \mathrm{Mulch} * \mathrm{Glyphosate} * \mathrm{Humic} + \mathrm{Condition}(\mathrm{scale}(\mathrm{logObs}))\\ &+\mathrm{Condition}(\mathrm{Timepoint})+\mathrm{Condition}(\mathrm{T0}_{-}\mathrm{MDS1})\\&+\mathrm{Condition}(\mathrm{T0}_{-}\mathrm{MDS2})+\mathrm{Condition}(\mathrm{T0}_{-}\mathrm{MDS3})). \end{align*}

Model reduction proceeded by sequentially removing nonsignificant higher-order interactions and statistical tests and plotting was completed as described above (Supplementary Table S4).

A bacterial and fungal genus-level differential abundance analysis was completed using ALDEx2 v1.41.0 to identify taxa enriched before (T0) or after treatment (T1, T2, T3) within each treatment group [62, 63] (Supplementary Methods S4; Supplementary Table S5).

### Field measurements analyses

Raw field data are available in Supplementary Table S6. Leaf nutrient data were used to assess treatment effects on temporal trait trajectories. The timepoint 0.5 (T0.5), where only mulch had been applied, served as the baseline. Relative change in individual nutrient concentrations was calculated for T1, T2, and T3 for each tree as: Relative Trait = (Trait_T_ − Trait_T0.5_)/Trait_T0.5_, where Trait_T_ represents the T1, T2, or T3 measurement. Relative trait values were fit to mixed-effects models using the lme4 (v1.1-37) package [64]. Data were visually assessed for outliers, which were removed. Removing the outliers did not impact the interpretation of the results; it only improved the model fit. Initial formula used for the mixed effects:


\begin{align*} \mathrm{lmer}(\mathrm{Rel.Trait}&\sim \mathrm{Mulch}*\mathrm{Glyphosate}*\mathrm{Humic}*\mathrm{Timepoint}\\&+ (1|\mathrm{Treat}_{-}{\mathrm{rep}})). \end{align*}

where the random effect “Treat_rep” accounts for repeated sampling of trees each year. Because HA contributed significantly to only three of the nine nutrient models, *post hoc* comparisons were simplified to the mulch × glyphosate treatment combinations within humic and nonhumic groups for each sampling timepoint (Mulch*Glyphosate|Humic|Timepoint). Candidate models of varying complexity were compared using Akaike’s Information Criterion corrected for small sample size (AICc) using MuMIn::model.sel() (v48.1), and the most parsimonious models, that included all three applications, were selected for inference. A type III ANOVA was performed using stats::anova(), to test for significance, followed by pairwise comparisons and significance tests using the emmeans (v1.10.2) and multicomp v1.4-28 packages [65, 66] with FDR adjustments (Supplementary Table S7). Data visualizations were generated using ggplot2 v3.5.1 [61].

Other field measurements, including transpiration and assimilation rates, average fruit weight, number of fruit, and weed dry weight measurements were fit to linear models, and the most parsimonious models were selected as described above, with the initial formula:


\begin{align*} \mathrm{lm}(\mathrm{Trait}\sim \mathrm{mulch}*\mathrm{glyphosate}*\mathrm{humic}_{-}{\mathrm{acid}} +\mathrm{year}). \end{align*}

Final models and results are included in Supplementary Table S8.

### Soil microbiota functional greenhouse experiment

A greenhouse experiment was conducted to examine plant responses to field-derived soil microbiota. Field soil was collected from each of the eight field treatment plots at the LREC on 8 October 2025. At this time, the mulch, glyphosate, and HA treatments had not been actively applied in ~14 months (field applications concluded August 2024). Soil was collected from three randomly selected trees per plot and pooled to generate one composite sample. For each tree, soil was sampled from a single side ~0.5 m from the trunk after removing the top 5 cm, to a depth of 25 cm. Approximately 0.5 L of soil was collected per tree (~1.5 L per plot). Tools were sterilized with bleach, and clean gloves were worn between plots. Samples were transported at room temperature and stored at 4°C. A portion of each soil sample was autoclaved twice for a 45-min liquid cycle (cooled between cycles) to create “heat-killed” microbiota controls. This created a total of 16 treatment groups (8 active, 8 heat-killed).

Carrizo rootstock seeds were purchased from Lyn Citrus Seed, Inc (Arvin, CA, USA) in September 2025. Prior to planting, seeds were soaked in sterile water at 28°C at 180 rpm for 24 h, to split seed coats. Seed coats were peeled to increase germination uniformity and planted the same day.

The greenhouse experiment was initiated on 7 November 2025. Double autoclaved 70:30 (v/v) coco coir (#766310389395, EnvironSoil, Irvine, CA) to perlite (#100552701, Vigoro, Lake Forest, IL) soil-like substrate was prepared. Each of the 16 soil microbiota treatments were homogenized in individual batches with the sterile soil-like substrate with 10% field-collected soil and 90% sterile substrate (v/v) [67]. This method has been shown to effectively transfer microbiota while reducing the effects of soil structure and nutrients. Sterile plastic cone pots (#CN-SS-SC-07B, Stuewe & Sons, Inc., Tangent, OR) were filled with 150 ml of field soil–inoculated substrate. One seed was planted per pot, ~20 mm deep, covered, and watered. Treatment pots (*n* = 256, 16 biological replicates per treatment group) were set up in a randomized block design. Pots were maintained under ambient greenhouse conditions. Plants were uniformly watered two times per week.

After 2.5 weeks, pots with multiple seedlings emerged were thinned by gently removing the additional seedling(s). In the case of multiple seedlings, the largest was left because these are more likely to be nuclear clones [68]. Pots that did not have seedling(s) emerge were noted and a seedling germinated in extra sterile substrate was transplanted into the pot.

After 14 weeks, the experiment concluded with measuring shoot height from the top of the soil to the top of the stem and recording seedling establishment. Then, plants were gently pulled from the soil, and soil adhering to the roots was shaken or pulled off. Whole plants were placed in paper envelopes and air-dried at room temperature for 6 days and then dried in an oven at 60°C for 24 h. After drying, dried roots and shoot mass were measured (Supplementary Table S9).

### Greenhouse experiment analysis

Plant establishment was analyzed as a binary response using a generalized linear model with a binomial error distribution and logit link. Firth’s bias-reduced logistic regression was implemented using the brglm2 package v1.0.1 [69, 70]. Fixed effects included mulch, glyphosate, and HA and their interactions. Significance of model terms was evaluated using a Likelihood Ratio Test. Pairwise comparisons, with FDR adjustment, were completed using the emmeans v1.11.2 [65] and multicomp v1.4-28 [66] packages.

Plant growth traits (dry root biomass, dry shoot biomass, and shoot height) were fit to mixed-effects models as described above. Initial formula used for the mixed effects:


\begin{align*} &\sim \mathrm{lmer}(\mathrm{trait}\sim \mathrm{mulch} * \mathrm{glyphosate}* \mathrm{humic}* \mathrm{Active.or.killed}\\&+(\mathrm{1|Block})). \end{align*}

where the random effect “Block” controls for variation in greenhouse conditions across experimental blocks and “Active.or.killed” represents whether the microbiota are active or autoclaved (heat-killed). Model reduction, statistical tests, and visualizations were completed using methods outlined above (Supplementary Table S10).

## Results

### Bacterial community composition was driven by application interactions, while fungal communities were primarily shaped by mulch

Before evaluating treatment effects on microbial community composition, we tested for differences among sampling timepoints (T0–T3) and field plots prior to field applications (T0) using a constrained ordination of Aitchison distances. Timepoint was a significant predictor of community composition across all communities, including bacterial root and rhizosphere and fungal root and rhizosphere (Supplementary Fig. S2; Supplementary Table S2). Pre-treatment (T0) fungal root and rhizosphere communities did not significantly differ among plots (Supplementary Table S3; Supplementary Fig. S3b and d). In contrast, bacterial root and rhizosphere communities exhibited significant compositional differences before treatments began (Supplementary Table S3; Supplementary Fig. S3a and c), indicating pre-existing variation among plots. These baseline differences were accounted for in subsequent analyses.

**Figure 2:**
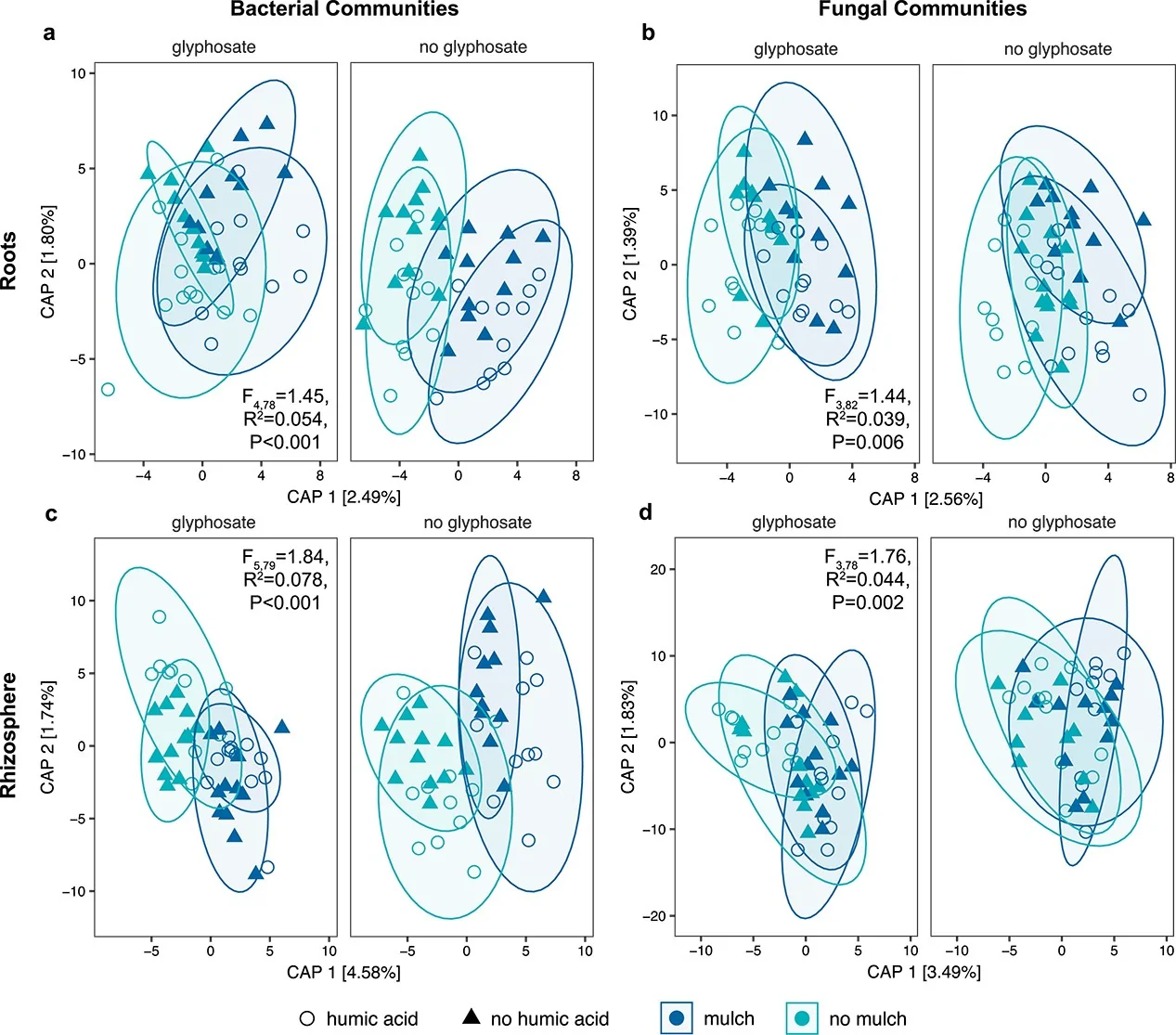
Field applications significantly impacted bacterial and fungal community composition after controlling for pretreatment variation. Constrained ordinations of Aitchison distances calculated for timepoints T1–T3 are shown for bacterial root (a; *n* = 89), fungal root (b; *n* = 92), bacterial rhizosphere (c; *n* = 91), and fungal rhizosphere (d; *n* = 88) communities. Ordinations were constrained by applications, including mulch (represented by color), humic acid (represented by shape), and glyphosate (represented in facet panels) while accounting for sequencing depth, timepoint, and pretreatment (T0) variation, and results from full-model ANOVA-like permutation (PERMANOVA) tests are reported within each panel. Ellipses represent 95% confidence intervals.

To test the effects of mulch, glyphosate, and HA on microbial community composition while accounting for pretreatment composition, we performed constrained ordinations of Aitchison distances, controlling for variation across years, sequencing depth, and the first three principal coordinates describing baseline (T0) community composition (Supplementary Table S4). Field applications significantly affected bacterial and fungal community composition across roots and rhizospheres (Fig. 2a–d; Table 2). The bacterial rhizosphere community was the most sensitive, with treatments explaining 7.8% of the community variation (Fig. 2b; Table 2).

**Table 2:** Canonical Analysis of Principal coordinates (CAP) ANOVA-like permutation test results, including the statistics for each term and interactions retained in the reduced model.

Compartment	Community	Model term	Df	Variance	F	P	Symbol
Root	Bacteria	Full model	4	189.17	1.4544	≤.001	***
		Mulch	1	65.09	2.0018	≤.001	***
		Glyphosate	1	39.19	1.2052	.073	.
		Humic	1	43.11	1.3259	.026	*
		Mulch:Glyphosate	1	41.78	1.2847	.043	*
		*Residual*	78	2536.27			
Rhizosphere	Bacteria	Full model	5	557.6	1.8443	≤.001	***
		Mulch	1	239	3.9526	≤.001	***
		Glyphosate	1	69.8	1.1547	.149	
		Humic	1	82	1.3554	.033	*
		Mulch:Glyphosate	1	87	1.4394	.012	*
		Glyphosate:Humic	1	79.8	1.3193	.031	*
		*Residual*	79	4777			
Root	Fungi	Full model	3	50.08	1.4387	.006	**
		Mulch	1	23.87	2.0568	.004	**
		Glyphosate	1	13.09	1.1284	.208	
		Humic	1	13.12	1.131	.207	
		*Residual*	82	951.49			
Rhizosphere	Fungi	Full model	3	135.59	1.7624	.002	**
		Mulch	1	73.25	2.8562	.002	**
		Glyphosate	1	39.13	1.5258	.067	.
		Humic	1	23.21	0.9051	.515	
		*Residual*	78	2000.31			

Symbols correspond to significance level based on *P*-value.

Across all four microbial communities examined, mulch application explained the largest proportion of variation in microbial community composition (Fig. 2a–d; Table 2). In fungal root and rhizosphere communities, mulch was the only application that significantly impacted microbiome composition (Table 2). In bacterial root and rhizosphere communities, mulch also explained the largest proportion of compositional variation, while HA had an additional significant effect. However, both bacterial communities also had significant mulch × glyphosate interactions, indicating that the effects of these applications were nonadditive and that the effect of mulch differed depending on glyphosate application (Fig. 2a and c; Table 2). The bacterial rhizosphere communities also showed a significant glyphosate × HA interaction. Together, these results indicate that bacterial community composition was shaped by combinations of applications, and in particular, glyphosate modifies the effects of mulch and HA, whereas fungal community composition was driven primarily by mulch alone. We also evaluated application effects on alpha diversity and found that responses were driven primarily by interactions among treatment (Supplementary Results S1; Supplementary Fig. S4; Supplementary Table S1).

### Mulch is the primary driver of taxonomic turnover in the root and rhizosphere microbiomes

**Figure 3:**
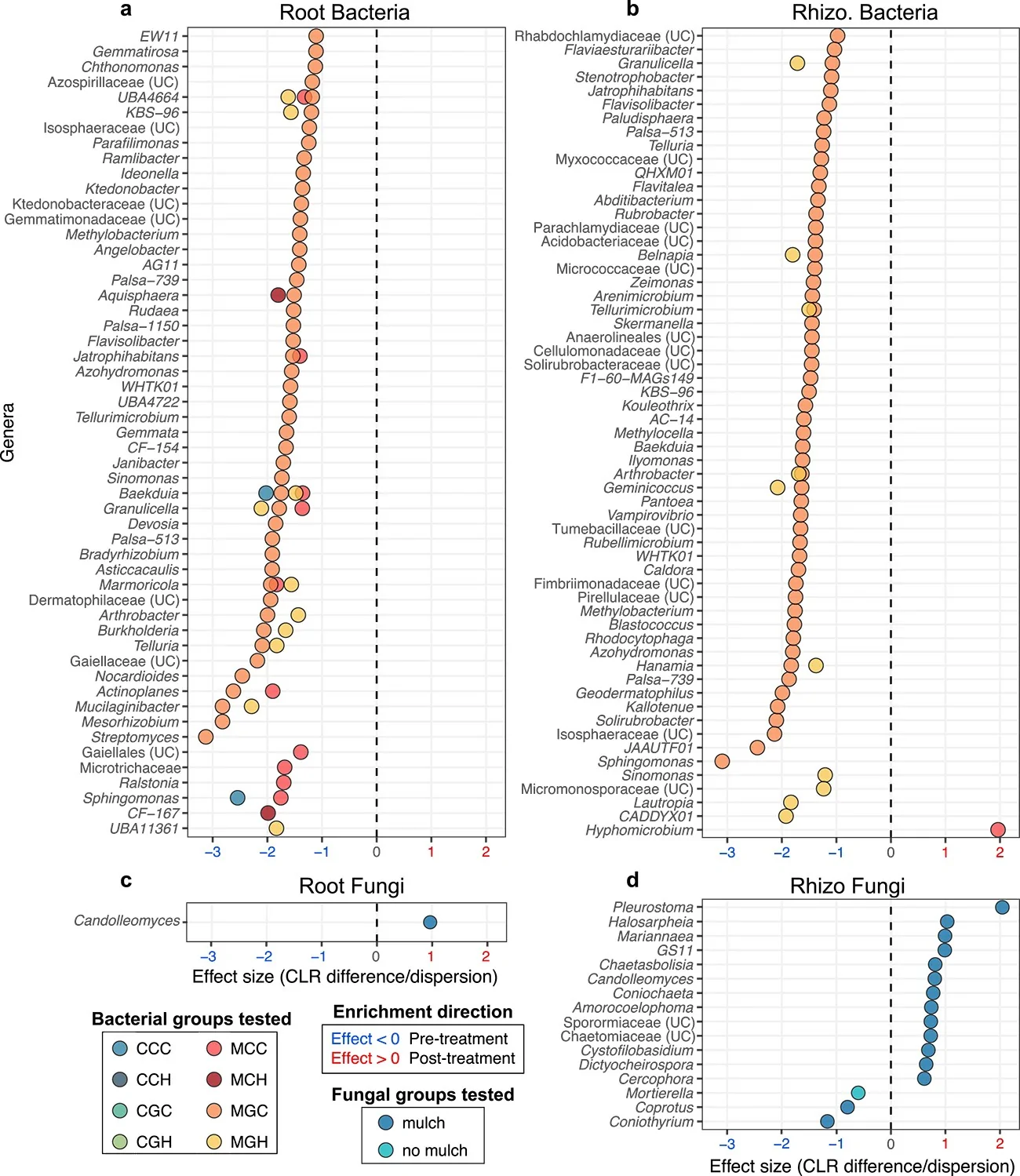
Wood mulch (alone or in combination) was the primary driver of microbial taxonomic turnover in citrus roots and rhizospheres. Genus-level differential abundance was assessed by comparing pretreatment (T0) with post-treatment samples (T1–T3) using ALDEx2 for bacterial root (a; T0, *n* = 32; T1–T3, *n* = 89), bacterial rhizosphere (b; T0, *n* = 32; T1–T3, *n* = 91), fungal root (c; T0, *n* = 32; T1–T3, *n* = 92), and fungal rhizosphere (d; T0, *n* = 31; T1–T3, *n* = 88) communities. Points represent effect sizes, calculated as the centered log-ratio abundance difference between post- and pretreatment samples divided by within-group dispersion. Negative values (blue) indicate greater relative abundance before treatments, whereas positive values (red) indicate enrichment after treatment; dashed vertical lines mark an absolute effect size of zero. For bacterial communities, colors denote the treatment group in which each significant genus was identified (CCC, CCH, CGC, CGH, MCC, MCH, MGC, and MGH), coded by position: mulch (M/C), glyphosate (G/C), and humic acid (H/C), where “C” indicates no application control. Because no fungal genera were significant within individual treatment groups, fungal samples were grouped by mulch treatment; colors therefore indicate mulch versus no-mulch comparisons. Bacterial genera included have an Benjamini–Hochberg-adjusted *P* < .05 and fungal genera have an Benjamini–Hochberg adjusted *P* < .10. Italicized *y*-axis labels indicate classified genera; when genus-level classification was unavailable, the family or order is shown, followed by “(UC)” for unclassified. Full statistical results are available in Supplementary Table S5.

To identify microbial taxa that changed in relative abundance following treatment, differential abundance analyses were performed comparing pretreatment (T0) to post-treatment (T1–T3) samples within each treatment group (Supplementary Table S5; Fig. 3). In bacterial root and rhizosphere communities, significant (*P* < .05) changes in genus abundance occurred almost exclusively in plots treated with mulch, either alone or in combination with glyphosate and/or HA (Fig. 3a and b). In particular, mulch × glyphosate (MGC) and mulch × glyphosate × HA (MGH) treatments drove bacterial genera differential abundance, again confirming that bacterial communities were largely shaped by application interactions. Only two bacterial genera, *Baekduia* and *Sphingomonas*, were significantly depleted in untreated control (CCC) roots after the start of experimental applications (Fig. 3a), suggesting that these taxa responded to annual environmental variation rather than treatment. The other 79 root and 64 rhizosphere bacterial genera were significantly depleted post mulch (alone or in combination) treatment (Fig. 3a and b). A methylotrophic bacterial genus, *Hyphomicrobium,* was the only genus enriched after mulch application (Fig. 3b) [71].

For fungal communities no individual treatment group contained significant genera enrichments pre- vs. post-treatment. Therefore, fungal samples were grouped by mulch treatment to increase statistical power, and the significance cutoff was relaxed to *P* < .1 (Fig. 3c and d). This approach identified one root genus (*Candolleomyces*) and eight rhizosphere genera enriched after mulch application, whereas *Coniothyrium* and *Coprotus* were depleted in the rhizosphere. Rhizosphere *Pleurostoma* was the most highly enriched fungal genus, following mulch application (Welch *t-*test, effect size = 2.04, *P* = 1.77 × 10^5^; Fig. 3d; Supplementary Table S5). Additionally, *Mortierella* was depleted post-treatment in the nonmulched plots, which likely reflects temporal environmental variation (Fig. 3d).

Collectively, except for three genera that changed in untreated control plots, no consistent microbial relative abundance shifts pre- versus post-treatment were detected in the absence of mulch, indicating that wood mulch was the primary driver of microbial taxonomic turnover during the experiment.

### Field applications altered temporal leaf nutrient trajectories

**Figure 4:**
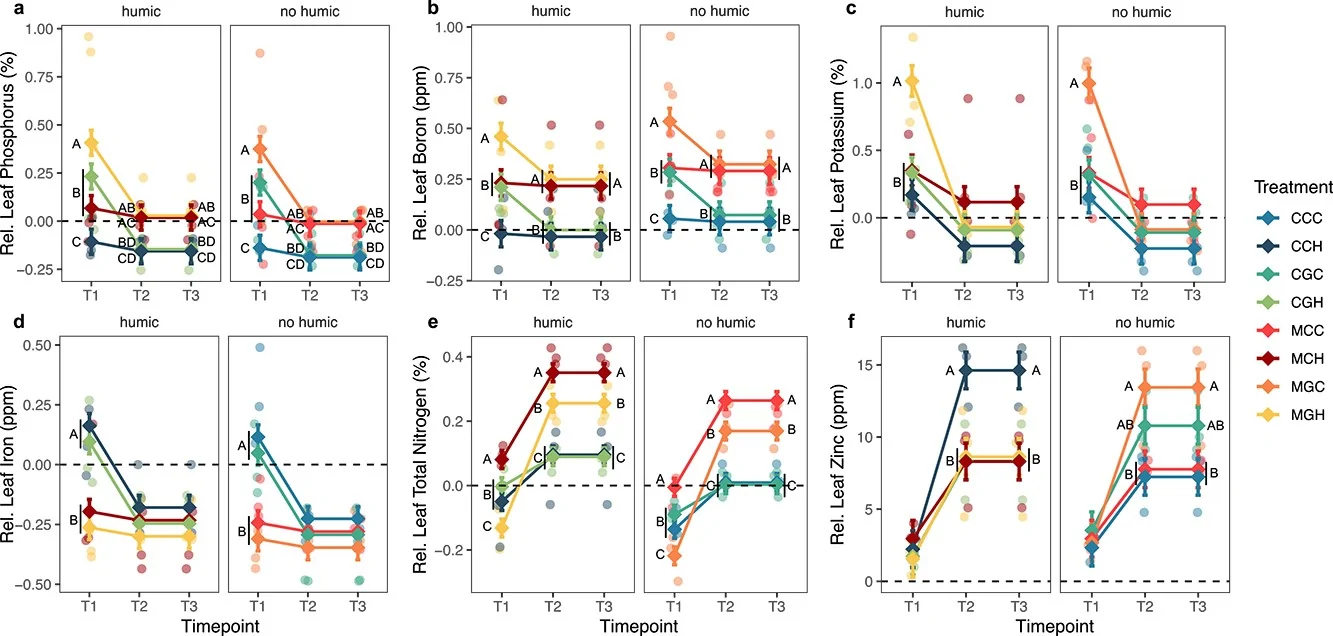
Experimental soil amendment applications altered temporal trajectories of citrus leaf nutrient concentrations. Relative changes in leaf phosphorus (a), boron (b), potassium (c), iron (d), total nitrogen (e), and zinc (f) were calculated for each tree relative to the T0.5 baseline, when only mulch had been applied [Relative Trait = (Trait_T_ − Trait_T0.5_)/Trait_T0.5_]. This analysis included *n* = 72 tree leaf samples. Diamonds and connecting lines represent estimated marginal means with bars showing standard error. Semi-transparent points show individual observations. Colors denote the eight treatment combinations (CCC, CCH, CGC, CGH, MCC, MCH, MGC, and MGH), coded by position: mulch (M/C), glyphosate (G/C), and humic acid (H/C), where “C” indicates the no application control. The dashed horizontal line indicates no change from baseline. Letters indicate pairwise group differences based on ANOVA, followed by a two-tailed Tukey *post hoc* test and FDR correction (*P* < .05). Pairwise comparisons among mulch × glyphosate treatment combinations within each humic-acid group and timepoint; groups sharing a letter were not significantly different. Timepoints with no letters indicated that there were no significant differences among those treatment groups at that timepoint. Final models and Type III ANOVA results are provided in Supplementary Table S7.

To determine whether the experimental applications produced measurable changes in tree phenotypes, we analyzed temporal trajectories of leaf nutrient concentrations (T1–T3) relative to the first post-treatment (mulch only) sampling timepoint (T0.5; Fig. 1). This approach allowed us to evaluate treatment-specific changes over time while minimizing possible differences in baseline nutrient status. Mixed-effects models identified significant treatment effects or treatment × timepoint interactions for eight of the nine nutrients examined, with relative calcium concentrations being unaffected (Supplementary Table S7). HA was a significant predictor for only three nutrients, affecting leaf total nitrogen as a main effect (ANOVA, *F*_1,19_ = 13.06, *P* = .0018), while copper and zinc exhibited significant HA interaction terms (Supplementary Table S7). Because HA only significantly influenced three of the nine nutrients examined, *post hoc* comparisons were simplified to focus on mulch × glyphosate treatment combinations within HA and no HA groups at each sampling timepoint (Fig. 4).

Phosphorus, boron, and potassium displayed similar treatment-specific trajectories, with trees treated with mulch and glyphosate in combination (MGC and MGH) having significantly higher relative concentrations at T1 (Fig. 4a–c). In contrast, mulch treatment significantly decreased relative iron concentrations in leaves at T1 (Fig. 4d). Relative leaf total nitrogen exhibited the strongest and most persistent treatment response. Trees receiving mulch (MCH and MCC) exhibited the largest relative nitrogen concentrations across all timepoints (T1–T3), followed by mulch × glyphosate treatments (MGC and MGH) at T2 and T3, whereas nonmulched treatments (CCC, CCH, CGC, and CGH) remained significantly lower at T2 and T3 (Fig. 4e). HA treated trees at T2 and T3 had significantly lower relative zinc concentrations if also treated with mulch and/or glyphosate (CGH, MCH, and MGH; Fig. 4f). Alternatively, non-HA-treated trees that received both mulch and glyphosate (MGC) exhibited significantly higher relative concentration of zinc than those that received only mulch (MCC) or no applications (CCC). Relative sodium, calcium, and copper concentrations did not have any significant pairwise contrasts (Supplementary Fig. S5).

Collectively, these results demonstrate that the experimental applications produced sustained, treatment-specific changes in citrus leaf nutrient trajectories, supporting that the experimental treatments altered tree phenotypes over time rather than reflecting pre-existing differences among treatment plots. Furthermore, application interactions played an important role in shaping leaf nutrient dynamics.

### Field applications significantly impacted tree productivity and weed biomass

**Figure 5:**
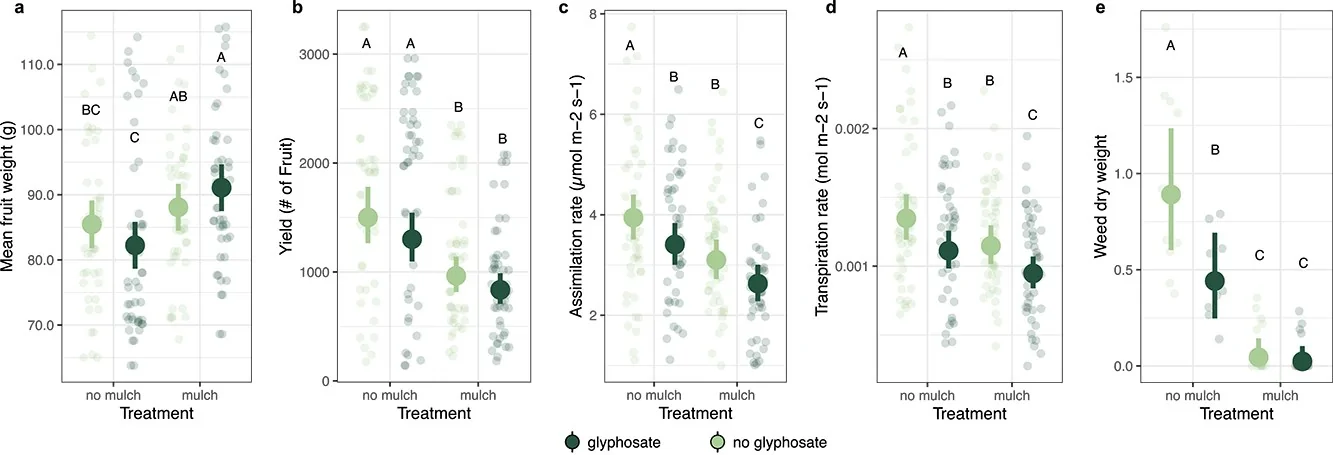
Mulch and glyphosate field applications significantly altered mature tree performance, productivity, and weed biomass. For all plots, points are estimated marginal means of biological replicates, error bars represent 95% confidence intervals. Letters indicate pairwise group differences based on ANOVA, followed by a two-tailed Tukey *post hoc* test and FDR correction (*P* < .05). (a) Average fruit weight per tree was measured in grams, and (b) yield was measured in total number of fruit and includes *n* = 192 tree samples across 3 years (T1–T3). (c) Carbon assimilation rate measured in μmol m^−2^ s^−1^ and (d) transpiration rate measured in mol m^−2^ s^−1^ includes *n* = 192 tree samples across 3 years (T1–T3). (e) Total aboveground weed dry weight was measured in kilograms and includes *n* = 48 plots across 2 years of sampling (T2 and T3). Colors represent glyphosate application.

Management-driven shifts in tree productivity and weed biomass were evaluated using linear models that included all post-treatment sampling years (T1–T3), with year treated as a fixed effect (Supplementary Table S8). HA was not a significant predictor of fruit weight, number of fruits, carbon assimilation, transpiration, or weed biomass variation and was therefore excluded from the final analyses. Mean fruit weight was significantly affected by mulch × glyphosate interaction (ANOVA, *F*_1,185_ = 4.73, *P* = .031; Fig. 5a) Trees that received mulch and glyphosate produced the heaviest fruit, which were an average of 8.9 g heavier than fruit from trees that were only treated with glyphosate (Fig. 5a). However, overall yield (number of fruit) was significantly lower for mulch-treated trees (ANOVA, *F*_1,182_ = 38.52, *P* = 3.56 × 10^−9^; Fig. 5b), with an average of 499 fewer fruits than nonmulched trees.

Mulch and glyphosate had an additive negative effect on tree carbon assimilation rate (Fig. 5c; ANOVA, mulch: *F*_1,186_ = 18.878, *P* = 2.29 × 10^−5^; glyphosate: *F*_1,186_ = 7.400, *P* = .007) and transpiration (Fig. 5d; ANOVA, mulch: *F*_1,185_ = 8.221, *P* = .005; glyphosate: *F*_1,185_ = 11.620, *P* = .0008). Although not quantitatively assessed, chlorosis (leaf yellowing) was also observed in the canopy of mulch-treated trees (Supplementary Fig. S6).

Weed biomass responded strongly to the field applications (Fig. 5e). Mulch significantly reduced weed biomass relative to unmulched plots (ANOVA, *F*_1,43_ = 93.214, *P* = 2.48 × 10^−12^). Glyphosate applied alone also reduced weed biomass, but produced an intermediate phenotype relative to mulch treatments (ANOVA, *F*_1,43_ = 6.868, *P* = .012). The combination of mulch and glyphosate did not differ significantly from mulch alone, indicating that mulch was the dominant driver of weed suppression.

### Management-driven soil microbiota changes altered plant performance in a greenhouse experiment

To test whether management-induced microbiota shifts likely contributed to the field-observed tree phenotypes, we conducted a greenhouse experiment using soil microbiota collected from each treatment plot ~1 year after the end of the experimental treatments. Soil microbiota were used to inoculate sterile substrate planted with Carrizo citrus rootstock seeds, the same rootstock variety used in the field experiment. For each soil treatment, an autoclaved (heat-killed) control was included to distinguish active microbial from abiotic soil effects.

Seedling establishment differed strongly between active and heat-killed microbiota treatments (Supplementary Fig. S7a). All autoclaved treatments exhibited 100% establishment, whereas active microbiota from mulch-treated and glyphosate × HA plots significantly reduced seedling establishment to only 6.3%–18.8%.

**Figure 6:**
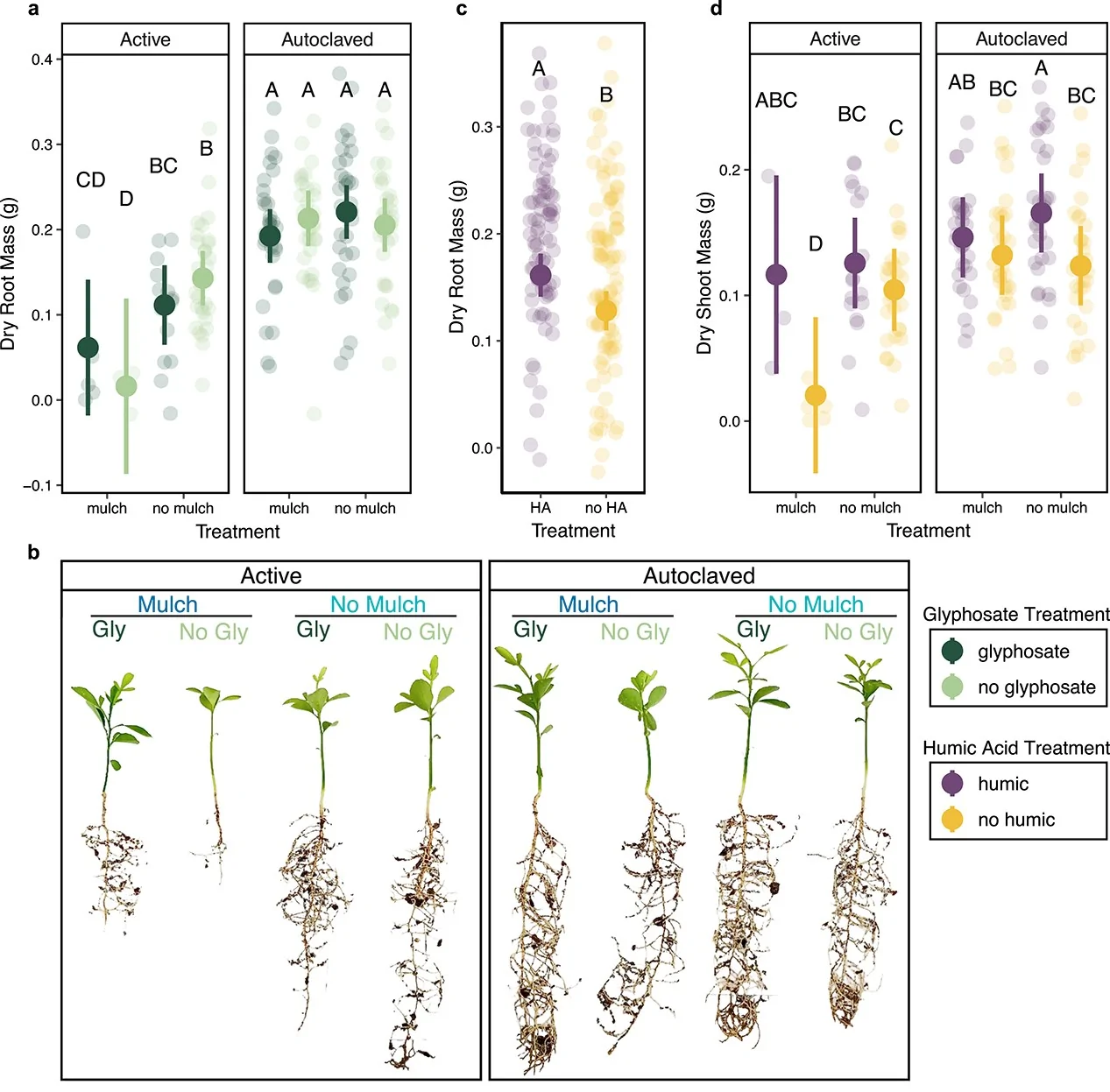
Field applications generated persistent soil microbiota functional changes that significantly impacted seedling root and shoot growth. (a) Root dry mass of seedlings grown with active or autoclaved soil microbiota inoculum from different field treatment plots. (b) Representative 14-week-old Carrizo rootstock seedlings from the corresponding soil microbiota inoculums. (c) Root dry mass of seedlings grown in soil microbiota originating from humic acid and no-humic acid plots, averaged across microbiota-activity and mulch–glyphosate treatments. (d) Shoot dry mass of seedlings grown with active or autoclaved soil microbiota inoculum from different field treatment plots. In panels (a), (c), and (d), large opaque points represent estimated marginal means, error bars indicate 95% confidence intervals, smaller semi-transparent points show individual biological replicates. Letters denote FDR-adjusted Tukey pairwise comparisons; groups sharing a letter did not differ significantly at *P* < .05. Analyses included *n* = 182 seedlings. Color indicates field treatments of the soil microbiota inoculum.

After 14 weeks, root and shoot traits were measured. Across treatments, seedlings grown with autoclaved microbiota inoculum developed significantly larger root systems than those grown with active microbiota (Fig. 6a–c; ANOVA, *F*_1,171_ = 82.47, *P* = 2.56 × 10^−16^). The effect of mulch depended on both microbial activity (active vs. heat-killed) (ANOVA, mulch × microbial activity: *F*_1,171_ = 7.67, *P* = .006) and glyphosate history (ANOVA, mulch × glyphosate × microbial activity: *F*_1,171_ = 4.03, *P* = .046; Fig. 6a,b). Root biomass did not differ among heat-killed microbiota treatment combinations. Seedlings grown with active microbiota originating from plots without mulch or glyphosate produced the largest roots. This indicates that management practices altered active soil microbiota functions in a way that negatively impacted citrus root development. Alternatively, soil originating from HA-treated plots increased seedling root biomass relative to no HA soils, regardless of whether microbiota were active or heat-killed (Fig. 6c; ANOVA, *F*_1,171_ = 13.25, *P* < .001). This suggests that HA primarily influences root growth directly, rather than through modifying microbiota functions.

Above ground growth responses were driven by mulch, HA, and microbial activity. Glyphosate treatment had no significant effects and was removed from final models (Supplementary Table S10). Shoot biomass and height were lower in seedlings grown in active microbial inoculum rather than heat-killed microbiota, but these effects depended on interactions with mulch and HA (Fig. 5d; Supplementary Fig. S7; ANOVA, *shoot biomass*: *F*_1,166_ = 6.16, *P* = .014, *shoot height*: *F*_1,166_ = 18.28, *P* = 3.20 × 10^−5^). Specifically, seedlings grown with autoclaved soil from HA-treated plots without mulch had significantly greater shoot biomass and height than plants that received autoclaved soil without HA (Fig. 6d; Supplementary Fig. S7b). Active microbiota originating from mulch-treated plots without HA produced significantly smaller shoot weights and heights (Fig. 6d; Supplementary Fig. S7b). Together, these results indicate that mulch altered active microbiota traits in a manner that suppressed above ground citrus growth, but HA moderated this effect.

## Discussion

Agricultural inputs act as ecological drivers that restructure soil microbial communities and alter plant–microbe interactions underlying crop productivity. Yet most management strategies were developed without considering their influence on soil and plant microbiota structure and function. Here, we show that common orchard practices reshape citrus root and rhizosphere microbiomes in ways that extend beyond compositional change to influence microbial community-level function and plant performance.

Wood mulch application emerged as the dominant driver of fungal community assembly and taxonomic enrichment, whereas bacterial communities responded strongly to combinations of mulch, glyphosate, and HA, primarily with depletions of taxa. These microbial responses suggest a shift toward a wood decomposition ecosystem. Wood mulch may have functioned as a fungal inoculum, transplanting wood-associated taxa into the rhizosphere [96], as well as introducing large quantities of complex organic carbon. Indeed, taxa belonging to genera that contain several known and putative fungal saprophytes associated with wood and leaf litter were enriched due to mulch treatment, including *Pleurostoma*, *Candolleomyces*, *Coniochaeta*, *Mariannaea*, *Cystofilobasidium*, and other members of Sordariomycetes (three genera) and Dothideomycetes (four genera) classes [72–77]. Deadwood metatranscriptomes are dominated by fungi, with 85% of the transcripts classified as fungal compared with only 13% bacterial, highlighting the central role of fungi in decomposing wood environments [78]. This putative shift toward fungal-dominated decomposition may have contributed to the depletion of 79 root and 64 rhizosphere bacterial genera after mulch (alone or in combination) application. This pattern may also reflect the strong suppression of herbaceous weeds by mulch, which removed living roots and exudates that could otherwise support these bacterial taxa. The only bacterial genus significantly enriched following mulch treatment, *Hyphomicrobium*, contains known methylotrophic species, consistent with evidence that saprotrophic fungi produce methane during wood decomposition that can support methylotrophic bacteria [78, 79].

The interactive effects of mulch and glyphosate may, in part, reflect mulch-induced changes in glyphosate persistence. Glyphosate degradation varies widely among soils, with reported half-lives ranging from days to several months, and is strongly influenced by the abundance and activity of glyphosate-mineralizing bacteria [80, 81]. Bacterial abundance and activity can be suppressed during wood decomposition [78], suggesting mulched plots may have reduced bacterial glyphosate degradation. Decomposing wood used as mulch can also absorb or bind glyphosate and, in some systems, more than double its half-life relative to bare soil [82]. HAs may further modify this response by binding glyphosate and increasing its persistence in soil, although they can also reduce its antibacterial activity *in vitro* [83–85]. These treatment interactions may explain the distinct patterns of bacterial genus depletion observed among mulch-containing treatment combinations (glyphosate and HA).

The field applications had a significant impact on leaf nutrient dynamics. We speculate that this is due to the effects of wood decomposition, altered microbial nutrient cycling, and changes in soil physicochemical conditions imposed by mulch-treatment associated shifts in root and rhizosphere microbial communities. Freshly applied wood mulch is high in carbon and low in nitrogen, creating strong microbial demand for nitrogen during early decomposition. However, as wood decomposition progresses fungal saprophytes can acquire nitrogen through associations with bacteria, and fungal carbon mobilization can support bacterial nitrogen fixation and accumulation within deadwood mulch [78, 86]. These processes could have progressively increased nitrogen availability beneath mulch and contributed to the persistently higher relative leaf nitrogen concentrations observed in mulch-treated plots. In addition, mulch provided strong weed suppression and, thus, reduced competition for nitrogen. We speculate that this weed suppression increased nitrogen availability for tree assimilation.

Despite increased concentrations of nitrogen and several other leaf nutrients, mulch and glyphosate were associated with reduced physiological performance (carbon assimilation and transpiration). Trees receiving mulch and glyphosate treatments produced fewer total fruit and had visible canopy chlorosis but had significantly heavier individual fruit. Lower crop load allows for greater resource allocation to each fruit but reduces the overall yield. Similar to other mulch-based field studies, the irrigation scheme was kept as a constant across all application plots to avoid introducing irrigation as a variable [87]. However, in practice, mulch application often increases soil water holding capacity. Consequently, mulched plots may have experienced excessive soil moisture, creating an environment conducive for performance-reducing microbes (pathogens) and limiting active iron (Fe^2+^) transport to leaves [88]. Indeed, we observed a decrease in relative leaf iron in mulch-treated plots during T1, coinciding with the development of canopy chlorosis and a decrease in photosynthetic activities. Iron deficiency-induced chlorosis is a common issue in citrus and other tree crops [89], and chlorotic leaves can have elevated nitrogen, phosphorus, and potassium concentrations negatively correlated with iron [90]. Thus, some of the apparent increases in macronutrients may reflect nutrient imbalance rather than improved nutritional status. Follow-up studies are warranted to determine if adding in irrigation as a treatment variable can capitalize on the weed suppression and macronutrient assimilation effects of mulch but reduce negative tree physiological effects (leaf chlorosis, reduced transpiration, carbon assimilation, and lower fruit yield). We also observed significant enrichment of taxa belonging to the *Pleurostoma* genus in mulch-treated rhizospheres. Some *Pleurostoma* species are pathogens of woody crops, such as olive, grape, and almond [76, 91–93]. Considering that the initial mulch used in this study was chipped almond wood, it could have served as a source of inoculum of *Pleurostoma* spp. Future studies of the *Pleurostoma*–citrus interaction are warranted, namely obtaining *Pleurostoma* isolates from the mulch-treated field plots and conducting Koch’s postulates to determine their pathogenicity in citrus.

A subsequent greenhouse experiment using soil from the treatment field plots provided evidence that treatment-induced microbial taxonomic shifts generated microbiota community-level functional changes that significantly influenced citrus seedling establishment and growth. Specifically, the negative effect of active microbiota from mulch-treated plots paralleled the negative physiological responses of field trees in mulched plots, which also experienced the largest shifts in root and rhizosphere microbiome structure. These findings support our hypothesis that orchard management practices can restructure microbial communities with consequences for crop performance. Additionally, the greenhouse experiment shows that microbiota composition and function were influenced by field treatments in ways that persisted even after applications ceased and soil was transferred to a new environment. Such microbiome legacy effects are increasingly recognized in soil ecology and can influence plant-microbe interactions, nutrient cycling, and plant responses to stress [4, 94]. However, microbiota were not the only driver of plant phenotypes. HA appeared to exert additional plant growth effects independent of microbial activity, which may reflect modifications in nutrient availability, root development, and/or plant hormone signaling [32].

These findings should be interpreted in light of the field experimental design, which was constrained by the costs of long-term mature tree maintenance, with each treatment combination being applied to a single orchard plot. Pretreatment sampling (T0) of every tree and repeated longitudinal sampling allowed us to account for baseline differences among plots and evaluate within-tree changes over time. The independently replicated greenhouse experiment provided complementary functional support for the field observations, while future replicated studies across additional orchards will help determine the magnitude and generality of these management effects.

Overall, this study highlights the importance of considering microbial ecology when evaluating agricultural management practices. Our findings demonstrate that orchard management inputs can restructure rhizosphere and root microbiomes and alter plant–microbe interactions. Understanding how management practices shape microbial community composition and function, can enable informed design of cropping management practices that promote beneficial plant–microbe interactions and minimize unintended microbiota disturbances.

## Supplementary Material

Supplementary_materials_ycag248

## Data Availability

The raw amplicon sequencing data supporting these findings have been deposited in the NCBI SRA repository and are associated with BioProject PRJNA1450731. Additionally, all field and greenhouse data and code used in the analyses are publicly available on GitHub and archived on Zenodo [https://doi.org/10.5281/zenodo.22149853 (microbiome reads processing); https://doi.org/10.5281/zenodo.22149932 (microbiome, field, and greenhouse analyses)].
