# Supplementary material for: Orchard management alters citrus root and rhizosphere microbiomes with functional consequences for plant performance
Source: ISME Commun. 2026 Sep 2;6(1):ycag248. doi: 10.1093/ismeco/ycag248 (PMC13600423; doi:10.1093/ismeco/ycag248)
Supplement: Supplementary file 1 [file supplementary_materials_ycag248.zip › Supplementary_materials_ycag248.pdf]

Supplementary Information for:

Orchard management alters citrus root and rhizosphere microbiomes with functional consequences for plant performance

Nichole Ginnan, Riley Jones, Jessica Wu-Woods, Tariq Pervaiz, Ashraf El-kereamy, Vanessa E.T.M. Ashworth, M. Imran Hamid, Emma K. Dawson, Sarah L. Strauss, Jason Stajich, Philippe Rolshausen, M. Caroline Roper

Corresponding Authors: M. Caroline Roper and Philippe Rolshausen
Emails: mcroper@ucr.edu and philrols@ucr.edu

This PDF file includes:

Supplemental Figures S1 to S7

Supplemental Tables S1 to S10

Supplemental Methods S1 to S4

Supplemental Results S1

Supplemental References

Supplemental Figures

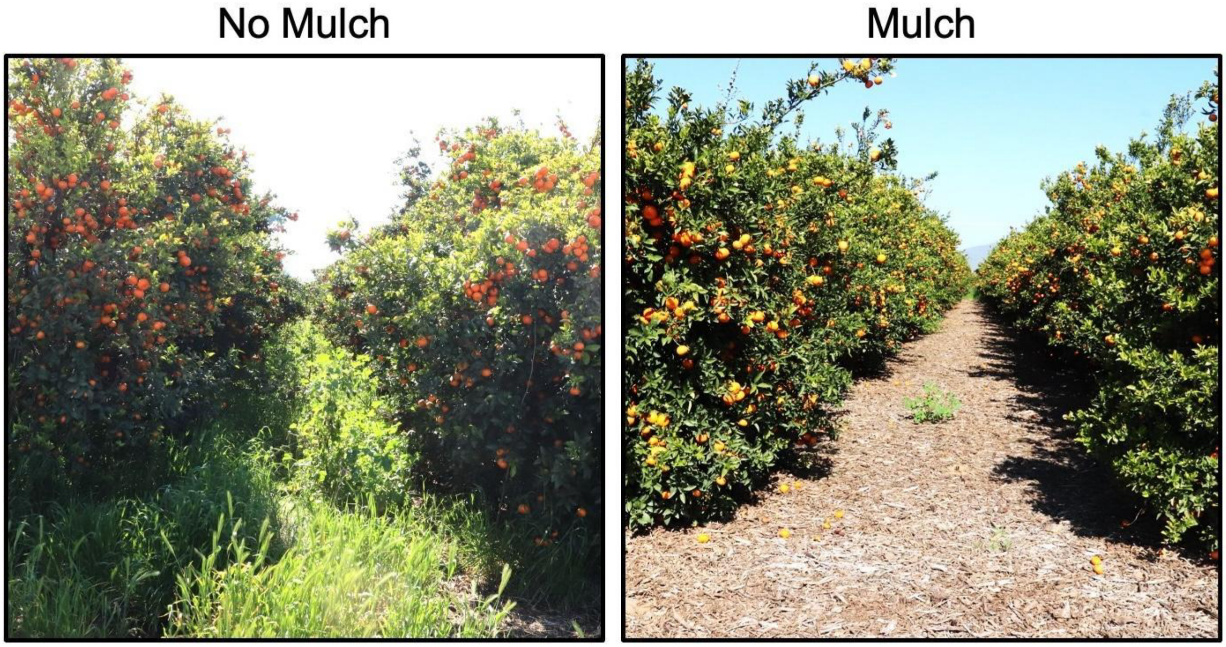

Supplemental Figure S1. Photographs of field plots without wood mulch and with wood mulch application.

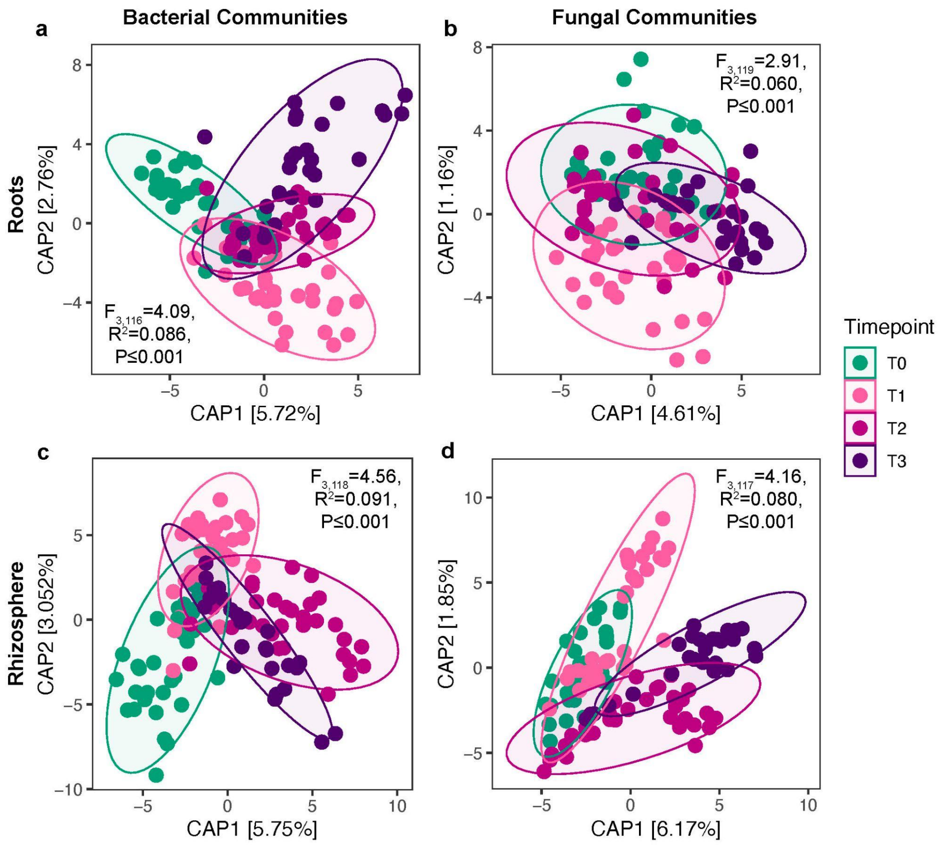

Supplemental Figure S2. Microbial community composition varied significantly across experimental timepoints. Constrained ordinations of Aitchison distances are shown for bacterial root (a; n=121), fungal root (b; n=124), bacterial rhizosphere (c; n=123), and fungal rhizosphere (d; n=122) communities. Points are colored by timepoint. Ordinations were constrained by experimental timepoint while accounting for sequencing depth, and results from full-model ANOVA-like permutation (PERMANOVA) tests are reported within each panel. Ellipses represent 95% confidence intervals.

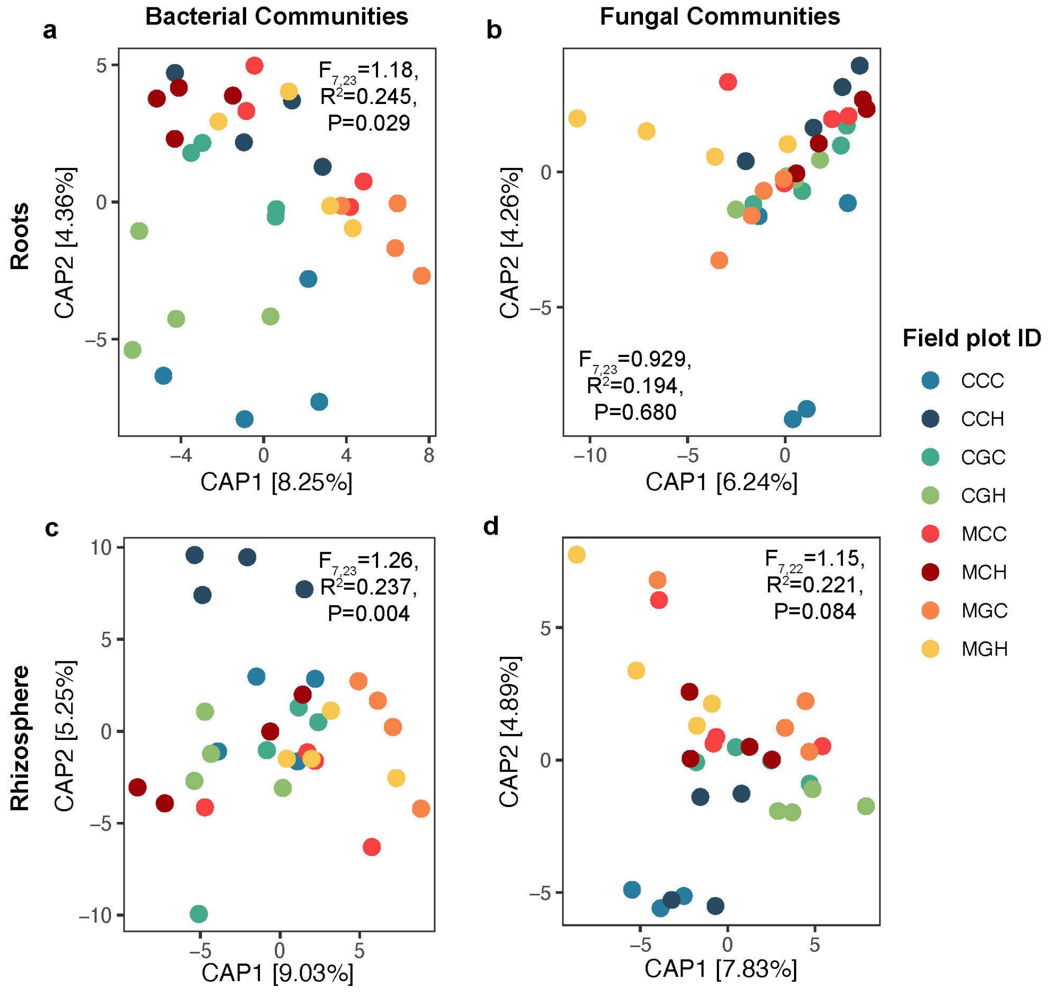

Supplemental Figure S3. Bacterial, but not fungal communities had significant compositional variation across plots, prior to treatments. Constrained ordinations of Aitchison distances calculated for timepoint zero (T0; pre-treatment) samples are shown for bacterial root (a; n=32), fungal root (b; n=32), bacterial rhizosphere (c; n=32), and fungal rhizosphere (d; n=31) communities. Ordinations were constrained by experimental field plot ID while accounting for sequencing depth, and results from full-model ANOVA-like permutation (PERMANOVA) tests are reported within each panel. Point colors denote the eight field plot IDs, which would later represent treatment combinations (CCC, CCH, CGC, CGH, MCC, MCH, MGC, and MGH), coded by position: mulch (M/C), glyphosate (G/C), and humic acid (H/C), where ‘C’ indicates no application control.

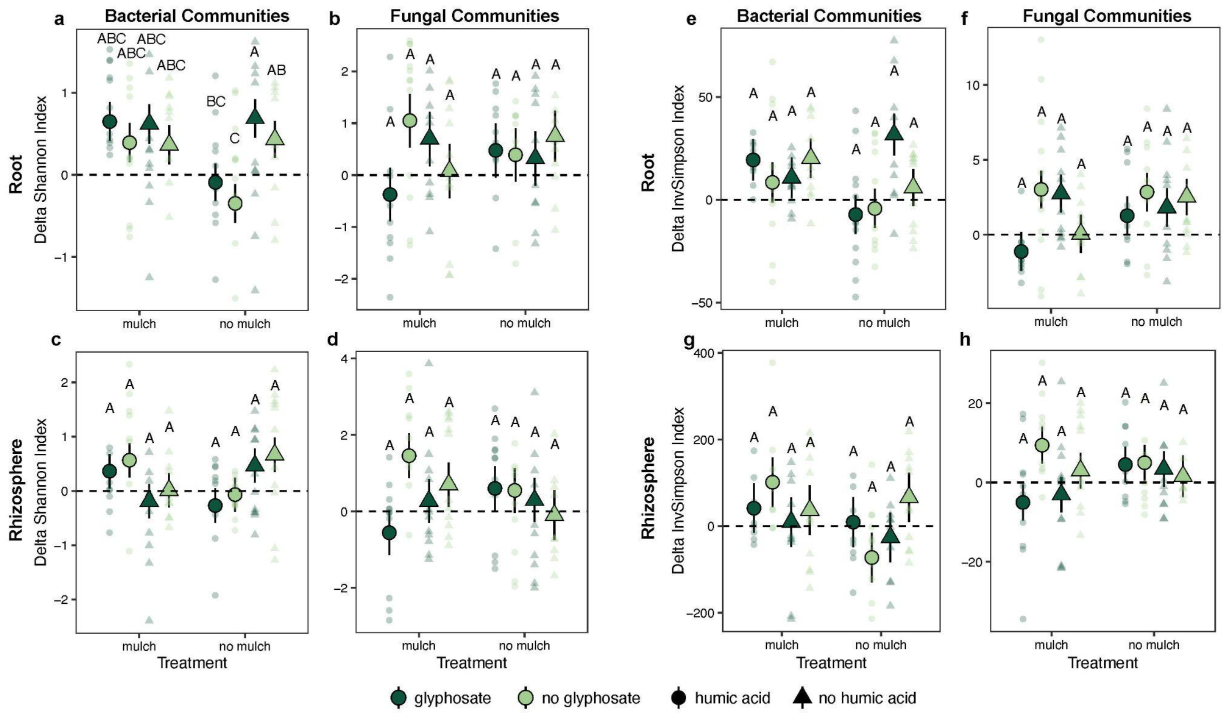

Supplemental Figure S4. Changes in bacterial and fungal alpha diversity following experimental treatments. Changes in Shannon diversity (Delta Shannon; a–d) and inverse Simpson diversity (Delta InvSimpson; e–h) were calculated for each tree by subtracting its pre-treatment value (T0) from each post-treatment measurement. Results are shown for bacterial root (a, e; n=89), fungal root (b, f; n=92), bacterial rhizosphere (c, g; n=91), and fungal rhizosphere (d, h; n=91) communities. Large symbols (circles and triangles) represent estimated marginal means with standard error bars, and semi-transparent points show individual data observations. Light green represents samples from plots that were not treated with glyphosate and dark green represents samples from glyphosate treated plots. Circles indicate humic acid treated samples and triangles are samples that were not treated with humic acid. For example, a light green triangle indicates no glyphosate and no humic acid. The dashed horizontal line marks the T0 baseline. Letters indicate pairwise group differences based on ANOVA followed by a two-tailed Tukey post hoc test and FDR correction ($P < 0.05$).

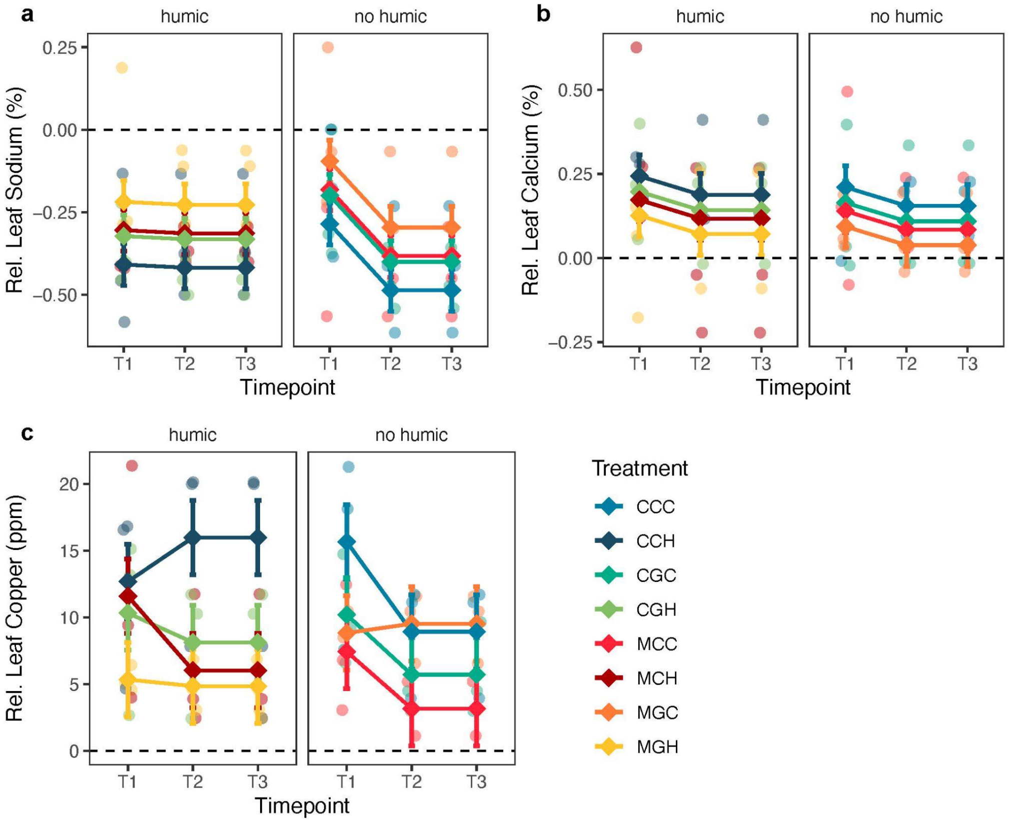

Supplemental Figure S5. Not all temporal trajectories on leaf nutrient concentrations were significantly impacted by mulch x glyphosate interactions. Relative changes in leaf sodium (a), calcium (b) and copper (c) were calculated for each tree relative to the T0.5 baseline, when only mulch had been applied [Relative Trait = $(Trait_{T} - Trait_{T0.5})/Trait_{T0.5}$]. This analysis included n=72 tree leaf samples. Diamonds and connecting lines represent estimated marginal means with bars showing standard error. Semi-transparent points show individual observations. Colors denote the eight treatment combinations (CCC, CCH, CGC, CGH, MCC, MCH, MGC, and MGH), coded by position: mulch (M/C), glyphosate (G/C), and humic acid (H/C), where ‘C’ indicates no application control. The dashed horizontal line indicates no change from baseline. Pairwise comparisons among mulch × glyphosate treatment combinations within each humic-acid group and timepoint; the absence of significance letters indicates that there were no significant differences among those treatment groups within those contracts. Final models and Type III ANOVA results are provided in Supplemental Table S7.

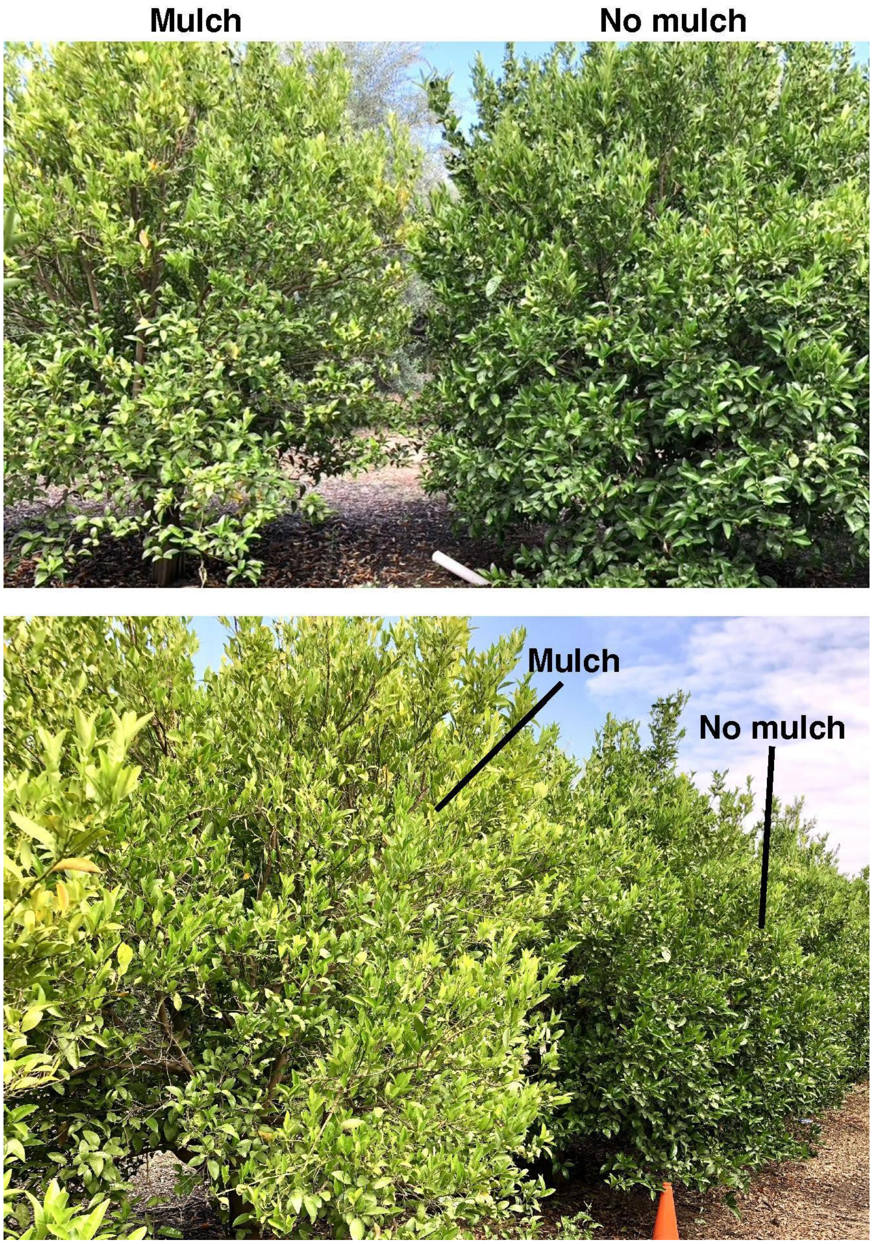

Supplemental Figure S6. Chlorosis was observed in mulch-treated plots.

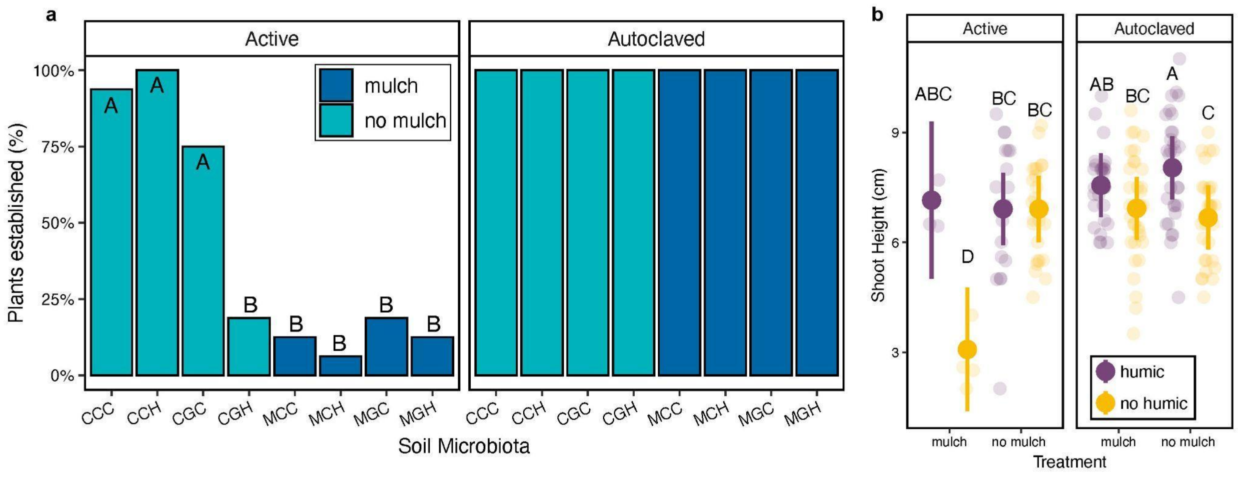

Supplemental Figure S7. Field applications alter soil microbiota functions that significantly impact seedling establishment and shoot height. Plots indicate the effects of treatment legacy on greenhouse seedling establishment (a) and shoot height (b). The large opaque points are estimated marginal means of biological replicates, error bars represent upper and lower 95% confidence intervals, and letters indicate pairwise group differences based on ANOVA followed by a two-tailed Tukey post hoc test and FDR correction ($P < 0.05$). The smaller semi-transparent points represent the raw data measurements of individual biological replicates. These analyses include n=182 biological replicate plant samples.

Supplemental Tables

Supplemental Table S1. Alpha diversity models and statistical results.
See supplemental table excel

Supplemental Table S2. Time point beta diversity models and statistical results.
See supplemental table excel

Supplemental Table S3. T0 beta diversity models and statistical results.
See supplemental table excel

Supplemental Table S4. Treatment (T1-T3) beta diversity models and statistical results.
See supplemental table excel

Supplemental Table S5. Pre- and Post-treatment differentially abundant microbial genera analysis results. The spreadsheet includes four tabs showing the ALDEx2 results for rhizosphere fungi, root fungi, rhizosphere bacteria, and root bacteria. Note, that the bacterial analyses also included eight archaeal genera that were amplified by the 16S rRNA primers. Analyses were completed at the genus-level ranking. Columns include treatment group being tested (treatment), taxonomic information (Phylum, Class, Order, Family, and Genus), mean centered-log ratio (CLR) abundance across all samples (CLR.abundance.all), CLR abundance during pre-treatment (pre-treatment.CLR.abundancer) and post-treatment (post-treatment.CLR.abundance) groups, between group differences (diff.btw), within group variation (dif.win), effect size (effect), proportion of Monte Carlo instances overlap (overlap), Welch's t-test expected (we.ep) and FDR corrected (we.eBH) P-values, Wilcoxon rank test expected (wi.ep) and FDR corrected (wi.eBH) P-values, the log scale of we.eBH (neglog10_q), significance

(sig), and the direction of taxa enrichment (direction). Significantly enriched taxa are highlighted in green.

See supplemental table excel

Supplemental Table S6. Field tree and weed data.

See supplemental table excel

Supplemental Table S7. Leaf nutrients models and statistical results.

See supplemental table excel

Supplemental Table S8. Other field data models and statistical results.

See supplemental table excel

Supplemental Table S9. Greenhouse experiment data.

See supplemental table excel

Supplemental Table S10. Greenhouse plant models and statistical results.

See supplemental table excel

Supplemental Methods

S1. Microbial DNA extractions from field samples.

Microbial DNA was extracted and purified using a high-throughput workflow implemented on a QIAcube HT robotic instrument (QIAGEN, Hilden, Germany). For citrus root material, 25 mg of lyophilized tissue was added to collection microtubes (#19560, QIAGEN) each containing one 3 mm-diameter tungsten carbide bead (#69997, QIAGEN). The frozen tissue was homogenized using a QIAGEN TissueLyser II for 2 min at 24 Hz in two rounds, first a dry round and then a second round in the presence of 500 µL Solution CD1 (DNeasy 96 PowerSoil Pro QIAcube HT kit, #47021, QIAGEN). The homogenate was centrifuged at 3214 *g* for 15 minutes and the supernatant was combined with 200 µL of Solution CD2 and centrifuged again. The supernatant was transferred to an S-block for DNA purification on the QIACube HT instrument. The *DNeasy*

96 PowerSoil Pro for PLANT_QCHT protocol was modified by replacing Solution CD3 with the viral lysis buffer (#19089, QIAGEN) DNA was eluted in 100 mL of Buffer EB (#19086, QIAGEN).

For rhizosphere sample DNA extraction, samples were defrosted, centrifuged for 8 minutes at 4000g, and 250 mg of the pellet was processed using the DNeasy 96 PowerSoil Pro QIAcube HT Kit (#12855, QIAGEN) following manufacturer's instructions.

Two positive control [75 µL of ZymoBiomics microbial community standard (#D6300, Zymo Research)] and two negative control (sterile Milli-Q water) samples were included in each 96-well plate. DNA concentrations were quantified using the QuantiFluor™ ONE dsDNA System (Promega, Madison, WI, USA) and measured on a BioTek Synergy HTX Multimode Reader (Agilent Technologies, Santa Clara, CA).

S2. Library preparation and amplicon sequencing of bacterial and fungal communities.

For bacterial amplicon sequencing libraries, 16S V4 rRNA gene amplification was performed using the 515F/806R primers [515F (Parada [1]), GTGYCAGCMGCCGCGGTAA); 806R (Apprill [2]), GGACTACNVGGGTWTCTAAT]. The 515F primers contained 12 basepair unique Golay barcode sequences and both the forward and reverse primers were purchased from IDT (IDT, Coralville, IA, USA) with Illumina adapter sequences attached, according the Earth Microbiome Project protocol [3, 4]. Additionally, Peptide nucleic acid (PNA) oligomers, including pPNA (GGCTCAACCCTGGACAG; PP01-25, PNABio, Thousand Oaks, CA, USA) and mPNA (GGCAAGTGTTCTTCGGA; MP01-25, PNABio, Thousand Oaks, CA, USA) clamps, were included in the PCR to block off-target amplification of citrus plant chloroplast and mitochondria 16S regions [5]. Each PCR reaction contained 10 uL Invitrogen Platinum Hot Start PCR Master Mix (2x) (#13000014, ThermoFisher, Waltham, MA, USA), 3 uL PCR-grade water, 3.75 uL of 5 uM mPNA, 3.75 uL of 5 uM pPNA, 2.0 uL of 2.5 uM 515F, 0.5 uL of 10 uM 806R, and 2.0 uL of template DNA for a total 25 uL volume reaction. For an amplicon size of ~390 bp, the PCRs had an initial denaturation at 94°C for 3 min; followed by 35 cycles of denaturation at 94°C for 45 s, annealing PNA clamps at 78°C for 10 s, annealing the primers at 50°C for 60 s, and extension at 72°C for 90 s; followed by a final extension at 72°C for 10 min. Each 96-well plate PCR included an additional two negative controls (PCR-grade water).

For fungal amplicon sequencing libraries, ITS1 region amplification was completed using the ITS1f/ITS2 primers [6] (ITS1f, CTTGGTCATTTAGAGGAAGTAA); ITS2, GCTGCGTTCTTCATCGATGC) based on the Earth Microbiome Project protocol [4, 7]. The ITS2 reverse primer contained the 12 bp unique Golay barcode sequences and both the forward

and reverse primers were purchased from IDT (IDT, Coralville, IA, USA) with Illumina adapter sequences attached. Each PCR reaction contained 10 uL Platinum Hot Start PCR Master Mix (2x) (#13000014, ThermoFisher, Waltham, MA, USA), 10.5 uL PCR-grade water, 0.5 uL of 10 uM ITS1, 2.0 uL of 2.5 uM ITS2, and 2.0 uL of template DNA for a total 25 uL volume reaction. For an amplicon size of ~250-600 bp, the PCRs had an initial denaturation at 94°C for 1 min; followed by 35 cycles of denaturation at 94°C for 30 s, annealing at 52°C for 30 s, and extension at 68°C for 30 s; followed by a final extension at 68°C for 10 min.

Bacterial and fungal PCRs were performed in duplicate and products were confirmed on a 1% agarose gel and then duplicates were pooled. Twenty-five µL of PCR product per sample were purified using the SequalPrep Normalization Plate Kit (ThermoFisher #A1051001) according to the manufacturer's recommendations. Then, 5 µL of each sample from one 96-well plate was pooled into one tube and underwent quality checks (QC) using a tapestation and qPCR at the UC Riverside Genomics Core Facility (UCR GCF). After QC, pooled samples were combined to have a final ratio of 2:3 16S:ITS and concentrated with Ampure XP Beads (Beckman Coulter #A63880) in a 1.8X bead ratio. The final pool followed the same QC pipeline prior to sequencing.

S3. Amplicon sequence processing.

For 16S reads, forward reads were discarded if they had more than 3 expected errors, otherwise they were truncated at 230 nucleotides; for reverse reads the parameters were 4 expected errors and 210 nucleotides. Error rates were estimated separately for forward and reverse reads based on a sample of the first 500 reads from each sequence file and then used to denoise and de-replicate reads using the standard DADA2 functions. Chimeric sequences were detected and removed using the ‘consensus’ procedure in DADA2. Each individual sample was processed in parallel, after which all the resulting ASV tables were merged. Finally, the taxonomy of each ASV was assigned using the DADA2 assignTaxonomy() function, which implements a naive Bayesian classifier, and the GreenGenes2 09.2024 release database [8].

For the ITS reads, forward reads were discarded if they had more than 3 expected errors, otherwise they were truncated at 220 nucleotides; for reverse reads the parameters were 4 expected errors and 220 nucleotides. Then, the ITS reads were processed in the same pipeline outlined for the 16S reads above. Finally, fungal taxonomy was assigned using the assignTaxonomy() function and the UNITE v.10 (02.19.2025 release) fungal database [9].

Post-processing, reads associated with plant contamination were removed from the ASV and taxa tables. Then, a threshold that discarded any ASVs that did not appear in at least five samples and had at least 20 reads was applied. ASVs with less than 100 total reads across all samples were removed to reduce rare ASVs and potential contaminants. Then, samples with <1000 reads were removed. The ZymoBIOMICS Microbial Community Standard (positive control) samples were evaluated and indicated that all taxa were present and contaminant ASVs represented less than 1% of the sample community. The positive and negative control samples were removed for downstream analyses.

S4. Microbial community analyses

Alpha diversity

Bacterial and fungal Shannon and inverse Simpson (InvSimpson) indices were calculated for the cleaned and prepared microbiome data using phyloseq::estimate_richness() [10]. Delta Shannon and Delta InvSimpson were calculated by subtracting timepoint zero (pre-treatment; T0) measurements from each of the post-treatment samples from the same tree (T1, T2, T3). Delta Shannon and Delta InvSimpson measurements and were fit to mixed-effects models using the lme4 (v1.1-37) package [11]Data was visually assessed for outliers, which were removed. Removing the outliers did not impact the interpretation of any of the results, only improved the model fit. The initial formulas used for the mixed-effects models:

lmer([Shannon OR InvSimpson] ~ Mulch * Glyphosate * Humic + z_logObs +
(1|Timepoint) + (1|Treat_rep)

where the random effects “Timepoint” represents the sample year and “Treat_rep” controls for repeated measure of the same trees each year, and “z_logObs” represents the z-scores of the log of the total observed sequence in that sample, which helps control for variation in sample sequencing depth. Candidate models of varying complexity were compared using Akaike's Information Criterion corrected for small sample size (AICc) using MuMIn::model.sel() (v48.1), and the most parsimonious models were selected for inference. A type III ANOVA was performed using stats::anova(), to test for significance, followed by pairwise comparisons and significance tests using the emmeans (v1.10.2) and multicomp v1.4-28 [12] packages [13] with FDR adjustments. All final models and ANOVA results are provided in Supplemental Table S1. Data visualizations were generated using ggplot2 v3.5.1 [14].

ALDEx2 details

Briefly, this program uses centered log-ratio (CLR) transformation of raw counts and Monte-Carlo sampling (mc.samples=200) to estimate the probability that a taxon is enriched. Then, we used ALDEx2::aldex.ttest(), which employs a Welch's t-test and Benjamini-Hochberg correction to test for significance. Results for all tested genera are listed in Supplemental Table S5. Effect size (magnitude of difference between two groups) was calculated using ALDEx2::aldex.effect(). Plots were created using ggplot2 [14].

Supplemental Results

S1. Field application interactions influenced changes in alpha diversity.

To assess bacterial and fungal alpha diversity responses to mulch, glyphosate, and humic acid (HA), we calculated the Shannon and inverse Simpson (invSimpson) Indices. To account for potential pre-treatment variation among plots, alpha diversity responses were evaluated as changes relative to each tree's baseline (T0) Shannon and invSimpson diversity indices. Treatment effects on Delta Shannon and Delta invSimpson were then assessed using mixed-effects models (Supplemental Table S1).

Overall, alpha diversity responses were driven by interactions among applications, rather than individual treatments. Although post-hoc pairwise comparisons were generally not significant after correction for multiple testing, the mixed-effects models consistently identified significant interaction terms (Supplemental Table S1).

Delta Shannon was significantly influenced by the interaction between mulch and HA in the bacterial root (ANOVA, $F_{1,31}$= 4.39, P = 0.044; Supplemental Fig. S4a) and bacterial rhizosphere (ANOVA, $F_{1,27}$= 11.46, P = 0.002; Supplemental Fig. S4c) communities. Root fungal community Delta Shannon was significantly affected by the three-way interaction among mulch, glyphosate, and HA (ANOVA, $F_{1,28}$ = 4.30, P = 0.048; Supplemental Fig. S4b), whereas the fungal rhizosphere Delta Shannon Index was not significantly impacted by the treatments.

In contrast, Delta invSimpson Index was comparatively stable across treatments (Supplemental Fig. S4e-h). No significant treatment effects were detected for bacterial root, bacterial rhizosphere, or fungal rhizosphere communities. Although fungal root Delta invSimpson was significantly influenced by glyphosate x HA interactions (ANOVA, $F_{1,26}$ = 5.62, P = 0.025; Supplemental Fig. S4).

Supplemental References

1. Parada AE, Needham DM, Fuhrman JA. Every base matters: assessing small subunit rRNA primers for marine microbiomes with mock communities, time series and global field samples. *Environ Microbiol* 2016; **18**: 1403–1414.
2. Apprill A, McNally S, Parsons R, Weber L. Minor revision to V4 region SSU rRNA 806R gene primer greatly increases detection of SAR11 bacterioplankton. *Aquat Microb Ecol* 2015; **75**: 129–137.
3. Earth Microbiome: 16S Illumina Amplicon Protocol. *Earth Microbiome Project*. https://earthmicrobiome.ucsd.edu/protocols-and-standards/16s/. Accessed 6 Mar 2026.
4. Caporaso JG, Lauber CL, Walters WA, Berg-Lyons D, Huntley J, Fierer N, et al. Ultra-high-throughput microbial community analysis on the Illumina HiSeq and MiSeq platforms. *ISME J* 2012; **6**: 1621–1624.
5. Lundberg DS, Yourstone S, Mieczkowski P, Jones CD, Dangl JL. Practical innovations for high-throughput amplicon sequencing. *Nat Methods* 2013; **10**: 999–1002.
6. Smith DP, Peay KG. Sequence depth, not PCR replication, improves ecological inference from next generation DNA sequencing. *PLoS One* 2014; **9**: e90234.
7. Earth Microbiome: ITS Illumina Amplicon Protocol. *Earth Microbiome Project*. https://earthmicrobiome.ucsd.edu/protocols-and-standards/its/. Accessed 6 Mar 2026.
8. McDonald D, Jiang Y, Balaban M, Cantrell K, Zhu Q, Gonzalez A, et al. Greengenes2 unifies microbial data in a single reference tree. *Nat Biotechnol* 2024; **42**: 715–718.
9. Abarenkov K, Nilsson RH, Larsson K-H, Taylor AFS, May TW, Frøslev TG, et al. The UNITE database for molecular identification and taxonomic communication of fungi and other eukaryotes: sequences, taxa and classifications reconsidered. *Nucleic Acids Res* 2024; **52**: D791–D797.

10. McMurdie PJ, Holmes S. phyloseq: an R package for reproducible interactive analysis and graphics of microbiome census data. *PLoS One* 2013; **8**: e61217.
11. Bates D, Mächler M, Bolker B, Walker S. Fitting linear mixed-effects models Usinglme4. *J Stat Softw* 2015; **67**.
12. Hothorn T, Bretz F, Westfall P. Simultaneous inference in general parametric models. *Biom J* 2008; **50**: 346–363.
13. Lenth RV. emmeans: Estimated Marginal Means, aka Least-Squares Means. *CRAN: Contributed Packages* . 2017. The R Foundation.
14. Wickham H. Ggplot2: Elegant graphics for data analysis. 2016. Springer, Berlin, Germany.
